# The Immunological Regulation Roles of Porcine β-1, 4 Galactosyltransferase V (B4GALT5) in PRRSV Infection

**DOI:** 10.3389/fcimb.2018.00048

**Published:** 2018-03-01

**Authors:** Lei Zhang, Jie Ren, Peidian Shi, Dong Lu, Chengxue Zhao, Yanxin Su, Lilin Zhang, Jinhai Huang

**Affiliations:** School of Life Sciences, Tianjin University, Tianjin, China

**Keywords:** porcine B4GALT5 (pB4GALT5), porcine reproductive and respiratory syndrome virus (PRRSV), glycoprotein 5(GP5), transcriptome analysis, immunological regulation

## Abstract

B4GALT5, also known as β-1, 4 galactosyltransferase V, is one of the members of β-1, 4 galactosyltransferase gene (B4GALT) family, which was concerned with embryonic development, tumor generation, other malignant diseases. In this study, we firstly cloned porcine B4GALT (pB4GALT5) from porcine alveolar macrophages, and predicted the structural domain and function of seven porcine β-1, 4 galactosyltransferase (I–VII) based on transcriptome analysis of PRRSV infected cells. Additionally, the upregulated porcine B4GALT5 expression was detected from PRRSV infected porcine alveolar macrophage (PAM) cells. The PRRSV proliferation were slightly inhibited in overexpression of pB4GALT5 transfected cells, the interaction of B4GALT5 and GP5 of PRRSV was firstly be detected by Co-IP, and the co-location between B4GALT5 and GP5 were also observed in golgi membranes by confocal microscopy. A significant increasing mRNA transcription, including inflammatory cytokines (IFN-α, IL-6, IL-18, IL-1β, TNF-α) and some cell surface glycosylated protein involved in antigen present (MHC-I/II), cell adhesion and migration (chemokine MCP-1 and receptor CCR2; LFA-1, ICAM-1) were upregulated in B4GALT5 overexpressed PRRSV infected cells. Our results demonstrated that the regulation of pB4GALT5 plays an important roles in PRRSV proliferation and modification function in viral infection cells. And these results will make achievements by supporting the research of latent mechanisms of β-1, 4 galactosyltransferase V in antiviral immunity.

## Introduction

B4GALT5, also known as β-1, 4 galactosyltransferase V, is one of β-1, 4 galactosyltransferase (B4GALT) family including seven members (Zhou et al., 2013). B4GALT is one of the most studied glycosyltransferases in recent years. These enzymes catalyze the shift of galactosyl group from UDP galactoside to N-acetylglucosamine or other glycosyl acceptor to determine the biosynthesis of different glycoconjugates (Zhang et al., 2017). Current research has shown that β-1, 4 galactosyltransferases are concerned with embryonic development (Kumagai et al., 2009), neurological development (Shen et al., 2003a,b), immune and inflammatory responses (Parker et al., 2016), tumor generation and development (Shirane et al., 2014), and other life activities (Wei et al., 2010) and diseases (Cartault et al., 2015). However, substrate specificity and distribution in different tissues of the members of the β-1, 4 galactosyltransferase family determine their functional differences (Fan et al., 1999; Ujita et al., 2000). At present, the functional aspect of β4Ga1T1 is the most studied, followed by β4Ga1T5.

Glycosylation is a common phenomenon in living organisms (Al-Ghouleh et al., 2012; Tavares-Carreón et al., 2016). Glycosyltransferases (glycosyltransferases, GT, EC 2.4.x.y) are specifically responsible for catalyzing the glycosylation and are the most diverse in nature (Terasaka et al., 2012; Schmid et al., 2016). The neck region of β4GalT5 contains multiple conserved N-glycosylation sites, it preferentially participates in N-linked galactosylation, but also catalyzes galactosylation of O-sugar chains (Furukawa, 2015). The phenomenon of viral protein glycosylation is widespread (DeVito et al., 2014; Bagdonaite et al., 2016), glycoproteins are necessary to the proliferation cycle of virus, recognition of host cells (Wasik et al., 2016) and viral fusion envelope-mediated with host cells (Khan et al., 2001).

Porcine reproductive and respiratory syndrome virus (PRRSV) is an *Arterivirus* of the family *Arterivirdae*. It has been determined that it is a single-stranded positive strand RNA with encapsulated membrane virus (Snijder et al., 1999). Sow breeding disorders, piglet respiratory symptoms and high mortality rate as the incidence characteristics (Bautista et al., 1996). The PRRSV genome is 15 kb long and contains eight Open Reading Frames (ORFs), ORF1 encodes the viral non-structural protein, ORF2~ORF7 encodes structural protein of the virus, the expression products of ORF2 ~5 are glycosylated envelope protein (Glycoprotein, GP 2~5), ORF6 and ORF7 encode membrane matrix proteins (M) and the nucleocapsid protein (N) (Conzelmann et al., 1993). Among these, GP5 is one of the highest degree of variations, but also is the main structure of PRRSV capsule glycoprotein, it is necessary to the infectivity and proliferation of PRRSV (Gao et al., 2014). Although the role of B4GALT5 in many different areas has been reported, it remains unknown whether it regulates the proliferation of capsular virus. In our research, we triumphantly acquired the porcine B4GALT5 (pB4GALT5) gene from porcine alveolar macrophages and compared evolutionary relationship between species based on amino acid sequence analysis. The effect of pB4GALT5 on proliferation of PRRSV was also surveyed. Our experiments would provide a basis for further revealing the function of GP5 protein glycosylation in viral replication and activation of immune responses.

## Materials and methods

### Cells and virus

Porcine alveolar macrophages (PAM) cell line 3D4/21 grown in RPMI-1640 medium (Gibco) added with 10% fetal bovine serum (FBS) (Biological Industries). Hela cells grown in Dulbecco's modified Eagle's medium (DMEM) added with 10% FBS, 100 U/ml penicillin and 10 μg/ml streptomycin sulfate in a 37°C, 5% CO_2_ incubator. The stock of PRRSV-JXwn06 with a titer of 10^4^TCID50/ml was used in this research.

### RT-PCR amplification of complete CDS of porcine B4GALT5 gene

A pair of oligonucleotide primer (listed in Table 1) was designed based on the coding sequence (CDS) of predicted pB4GALT5 sequence (NCBI Reference Sequence: XR_002340099.1). Intracellular RNA were abstracted using TRIzol from TaKaRa. RAN were reverse transcript by reverse transcription kit (TaKaRa). The cycler program was consisted with 95°C for 5 min, then 30 cycles at 95°C for 30 s, 56°C for 30 s and 72°C for 1.2 min using 2 × PCR Solution Premix Taq™ from TaKaRa.

**Table 1:** Primers used in amplification and plasmid construction.

**Primer^a^**	**Sequence (5′-3′)^b^**	**Product size**	**Use**
1F	CG*GAATTC*AATGCGCGTCCGCCGGGGG	1,167bp	Flag-B4GALT5
1R	GC*TCTAGA*TCAGTACTCGGTCACCTGAG		
2F	CG*GAATTC*ATGCGCGTCCGCCGGGGGC	1,167bp	B4GALT5-DsRed
2R	GG*GGTACC*GTGTACTCGGTCACCTGAGC		
3F	CG*GAATTC*ATGTTGGGGAAATGCTTG	603bp	GP5-Myc
3R	CC*CTCGAG*AGGACGACCCCATTGTTC		
4F	CC*CTCGAG*CTATGTTGGGGAAATGCTTG	603bp	GFP-GP5
4R	GG*GGTACC*CTAAGGACGACCCCATTG		

a *F/R meant upstream/downstream primers*.

b *Dash indicated restriction sites*.

### Bioinformatics analyses and sequence alignment

Bioinformatics analyses include protein structure analyses and functional domain analyses. Transmembrane region analyses was conducted using online software CBS Prediction Servers (http://www.cbs.dtu.dk/services/TMHMM-2.0/). Protein secondary structure was predicted using the PHYRE 2 online soft (http://www.sbg.bio.ic.ac.uk/phyre2/html/). Then, we imitated 3D structure using online website (http://zhanglab.ccmb.med.umich.edu/I-TASSER/). I-TASSER was an abbreviation of Iterative Threading AS Assembly Recession. It was a hierarchical approach to forecast structures and functions of multiple proteins. It would recognize the structural template from the Protein Data Bank (PDB) through the multi-threaded method LOMETS, which built a full-length atomic model through iterative template fragment simulated atomic assembly. The 3D model was then threaded through the BioLiP for ligand-protein binding database to insight into the function of the target protein. Subsequently, I mapped its critical domains using PyMOL software (https://pymol.org/2/). Amino acid sequences were aligned using the DNAMAN program (http://www.lynnon.com/dnaman.html). Meanwhile, sequence similarity analyses were conducted using the MEGA5.0 (http://www.megasoftware.net).

### Plasmid constructions

The cDNA of pB4GALT5 was amplified and sub-cloned into the vector pFlag-CMV-2 (Sigma-Aldrich) and pDsRed-Monomer-N1 (Clontech) to generate the fusion plasmid of B4GALT5-DsRed and Flag- B4GALT5. The GP5 gene of PRRSV JXwn06 was amplified and cloned into the vector pcDNA3.1 (Invitrogen) and pEGFP-C1 (Clontech) to generate the plasmid of GP5-Myc and GFP-GP5. Above constructs were verified by sequencing and the sequences of primers were supplied in Table 1.

### Transcriptome sequencing and analysis

3D4/21 cells were cultured in 10 cm culture dish at 10^6^ cells/ml. After cells were grown to monolayer and were incubated with PRRSV at 0.5 MOI for 24 h. And then we washed twice with 1 × phosphate-buffered saline (PBS) and add 1 ml of Trizol to lyse cells. The cells were then transferred to a cryopreserved tube and sent to Guangzhou GENE DENOVO Company for transcription sequencing.

The specific operation flow is as follows. After extracting the total RNA, the mRNA was enriched with the magnetic beads with Oligo (dT). To add the fragmentation buffer so that mRNA becomes a short segment. And then the first strand was synthesized by random hexamers using fragmented mRNA as template, the addition of buffer, dNTPs, RNase H and DNA polymerase I synthesis cDNA secondary chain. Following, the acquired cDNA was purified and eluted by QiaQuick PCR kit and EB buffer. Next, to add base A, and sequencing linkers after the ends were repaired. And then the purpose fragments were recovered by agarose gel electrophoresis, and PCR amplification was carried out to complete the whole library preparation. The constructed library was sequenced with Illumina HiSeqTM 2500.

The obtained transcriptome data were analyzed using online website (http://www.omicshare.com/tools/Home/Soft/search) and drawn a heat map to show the differential expression of the gene.

### Quantitative real-time PCR (qRT-PCR)

The comparatively quantification of gene expression level was measured by qRT-PCR run on ABI 7500 Real-Time PCR system, the thermal cycler program consisted of 95°C for 10 min, then 40 cycles at 95°C for 15 s, 55°C for 30 s and 72°C for 30 s using DBI Bioscience-2043 Bestar SybrGreen qPCR mastermix. All the primers were shown in Table 2.

**Table 2:** Primers used in quantitative real-time PCR.

**Gene**	**Accession no**.	**Primer sequence (5′-3′)**	**Product (bp)**	**Tm (°C)**
B4GALT5	XM_003134490.4	AACAGTTTCGGAAAATCAATGGC	196	56.4
		TTCCTCAGCAGGGCATACCTT		
GP2a	JX317649.1	CTACCCATGCTGCACAACC	260	55.4
		AGAAAAGTTGCCCCTAACCAG		
GP3	JX317649.1	CACCTTGCCTCGCCATGACA	292	57.4
		ACTGAGAACTTTTGCGAATCGT		
GP4	JX317649.1	CTCTCCGACGATTCGCAAA	275	55.6
		CATGATCGACCACTAAGGAGC		
GP5	JX317649.1	CCAGCCATTTCCTTGACACA	285	56.0
		ACAACTCTCTTGAGGTCGAT		
N	JX317649.1	GCCCAGCAAAACCAGTCC	256	58.4
		GCGTTGGCAGACTAAACT		
β-actin	DQ452569.1	GAATCCTGCGGCATCCACGA	230	55.0
		CTCGTCGTACTCCTGCTTGCT		
MHCI	NM_001097431	CACGGCGGCTCAGATCACCA	288	58.6
		GTCTCCACCAGCTCCATGTCC		
MHCII	NM_001113695	ATTAAACGTTCCAACAACACCG	296	56.3
		TTATCCAGGCCCCAGTGCTC		
IFN-α	JQ839262.1	TGAGAGCCTCCTGCACCA	267	56.0
		CTTCTGCCCTGACGATCTC		
IL-6	AF518322.1	TACTGGCAGAAAACAACC	294	58.3
		TCTGTGACTGCAGCTTATC		
IL-18	AF191088.1	TACACTTTACTTTGTAGCTGA	305	56.5
		TAGACATTTTCTTACACTGC		
IL-1β	M86725.1	AGAGCATCTTTTCATCCGTCT	253	57
		GTCACAGGTATCTTGTCATCG		
TNF-α	X57321.1	GCTGCCCTTCCACCAACGTTT	203	58.4
		CTCGGCACTGAGTCGATCATCC		
MCP-1	AJ311717.1	CTCCTGTGCCTGCTGCT	282	55
		TTCAAGGCTTCGGAGTTT		
CCR2	AB119271.1	AACATTCTGGTTACGCCTGT	124	52.7
		ATTCCCGAGTAGCAGACG		
LFA1	NM_213908.1	GAAGTTCGACACCGGCCCCTT	254	63.4
		TGCCCACGAGCACGACTCCC		
ICAM1	NM_213816.1	TCCTGTATGGACCCCGGCTA	256	58.0
		TGGATCACGTTCACGACCAC		

The data processing employed the relative cycle threshold (CT), that is 2^−ΔΔCt^ method, and ΔΔCt was calculated according to the following formula:

$$\begin{array}{l} \Delta {\text{Ct}}_{\left(\text{experimental group}\right)}=\text{X gene Mean Ct }-\beta -\text{actin Mean Ct};\\ \Delta {\text{Ct}}_{\left(\text{control group}\right)}=\text{X gene Mean Ct }-\beta -\text{actin Mean Ct};\\ \Delta \Delta {\text{Ct}}_{\left(\text{experimental group }-\text{ control group}\right)}=\Delta {\text{Ct}}_{\left(\text{experimental group}\right)-}\Delta {\text{Ct}}_{\left(\text{control group}\right)}\\ \text{Relative mRNA expression of the X gene }=\;2^{-\Delta \Delta \text{Ct}\left(\text{experimental group }-\text{ control group}\right)}\\ \left(\text{X gene means common name of detected gene}\right) \end{array}$$

The calculation results were the relative expression amount of the genes, so as to eliminate the interference of the irrelevant foreign protein expression as much as possible and reduce the experimental error.

### Flow cytometry assay

To determine the expression of pB4GALT5, 3D4/21 cells were dealt with PRRSV strain JXwn06 at 0.5 MOI or mock infected with DMEM. Twenty four hours after inoculation, the 3D4/21 cells were washed twice with PBS to suck away dead cells, separated from the dishes with trypsin at 37°C for 5 min, and then rinsed three times with cold PBS. The cells were used for staining with murine polyclonal antibody of B4GALT5 at 4°C for 30 min and then incubated with anti-mouse IgG with FITC (BD pharmingen™) (1:200) for 30 min at 4°C. By flow cytometry, a total of 2 × 10^4^ cells were detected, and fluorescence intensity was viewed as the expression of pB4GALT5.

### TCID_50_ assay in regard to PRRSV titers

PRRSV titers were evaluated by a doubling dilution assay and the titers were denoted as the 50% cells culture infective dose (TCID_50_)/ml through the Reed-Muench method (Wang et al., 2011). In short, 3D4/21 cells were scattered in 96-well plates, following, incubated with serial 10-fold dilutions (100 μl) of the primary PRRSV and set two column repeats for each dilution. Allow virus to adsorb to cells at 37°C for 1 h, then, the virus inoculum was sucked away, and RPMI-1640 adding 2% FBS. Cells were incubated for 48–72 h. Virus titers were calculated by determining the dilution of 50% cells culture infective dose that reveal cytopathic appearances.

### Small RNA (siRNA) interfering assay

For evaluating regulation of B4GALT5 on proliferation of PRRSV and expression of immune-related molecules, a siRNA assay was carried out by utilizing siRNA for B4GALT5 (siB4GALT5) and negative control (NC) that were synthesized though GenePharma (Tianjin, China; Table 3). The siRNAs were transfected at 50 nmol using Lipofectamine3000 (Invitrogen) for 24 h. Then, cells were dealt with the PRRSV at 0.5 MOI for 24 h. Quantitative real-time PCR and Western blot analyses were performed to confirm the expression levels.

**Table 3:** Primers used in small RNA interfering assay.

**Gene**	**Accession no**.	**Primer sequence (5′-3′)**
Negative control	NC	F:UUCUCCGAACGUGUCACGUTT
		R:ACGUGACACGUUCGGAGAATT
siB4GALT5-1	Sus-236	F:GCGUGAACGACUCAGACUATT
		R:UAGUCUGAGUCGUUCACGCTT
siB4GALT5-2	Sus-448	F:GGAGGUCACUGGAAGCCAUTT
		R:AUGGCUUCCAGUGACCUCCTT

### Confocal immunofluorescence assay

Flag-B4GALT5 and GP5-myc were transfected into Hela cells by Lipofectamine™ 2000 (Invitrogen). 4% paraformaldehyde fixed cells for 30 min, and then, respective primary antibodies dealt with cells for 1 h. Tris buffered saline-tween-20 (TBST) washed twice, anti-rabbit IgG- FITC or anti-mouse IgG- Cy5 dealt with cells for 2 h under indoor temperature. Nuclei were stained with DAPI. At last, the photographs were visualized by a PerkingElmer (America) laser confocal imaging analysis system after nuclear staining.

In addition, 3D4/21 cells were co-transfected with GFP-GP5 and B4GALT5-DsRed, GFP-Golgi marker (received from Tianjin Medical University epigenetic laboratory) and B4GALT5-DsRed. The cells at 36 h post-transfection were fixed and cellular nuclei were stained with DAPI. The rest of the treatment was the same as above.

### Co-immunoprecipitation and western blot

GP5-myc and Flag-B4GALT5 were transfected for 48 h in 3D4/21 cells. Following, they were lysed with lysis buffer (APPLYGEN, Beijing, China). To separate cell lysates at 13,000 rpm for 8 min though centrifugation. Then, mouse anti-Flag mAb Sepharose beads (Sigma) added to supernatants and incubated 2 h, the sediments were gathered and the beads were washed twice using lysis buffer. Proteins were transferred from SDS-PAGE to a piece of nitrocellulose membrane (NC) (ExPro). The membrane was dealt with mouse anti-Myc antibody, and goat anti-mouse IgG with horseradish peroxidase, and detected though chemiluminescence detection kit (Thermo Scientific, USA) and was viewed though Gel Imaging System (BIO-RAD, USA).

### Statistical analysis

Above experiments were carried out with three independent replicates. Results were dealt with GraphPad Prism software using *t*-test. Error was signed as the standard error of the mean (SEM). Differences in data were taken for be statistically significant if the *P*-value was less than 0.05 (^*^p < 0.05; ^**^p < 0.01; ^***^p < 0.001).

## Result

### Porcine B4GALT5 expression was up-regulated in 3D4/21 cells by PRRSV infection

**Figure 1:**
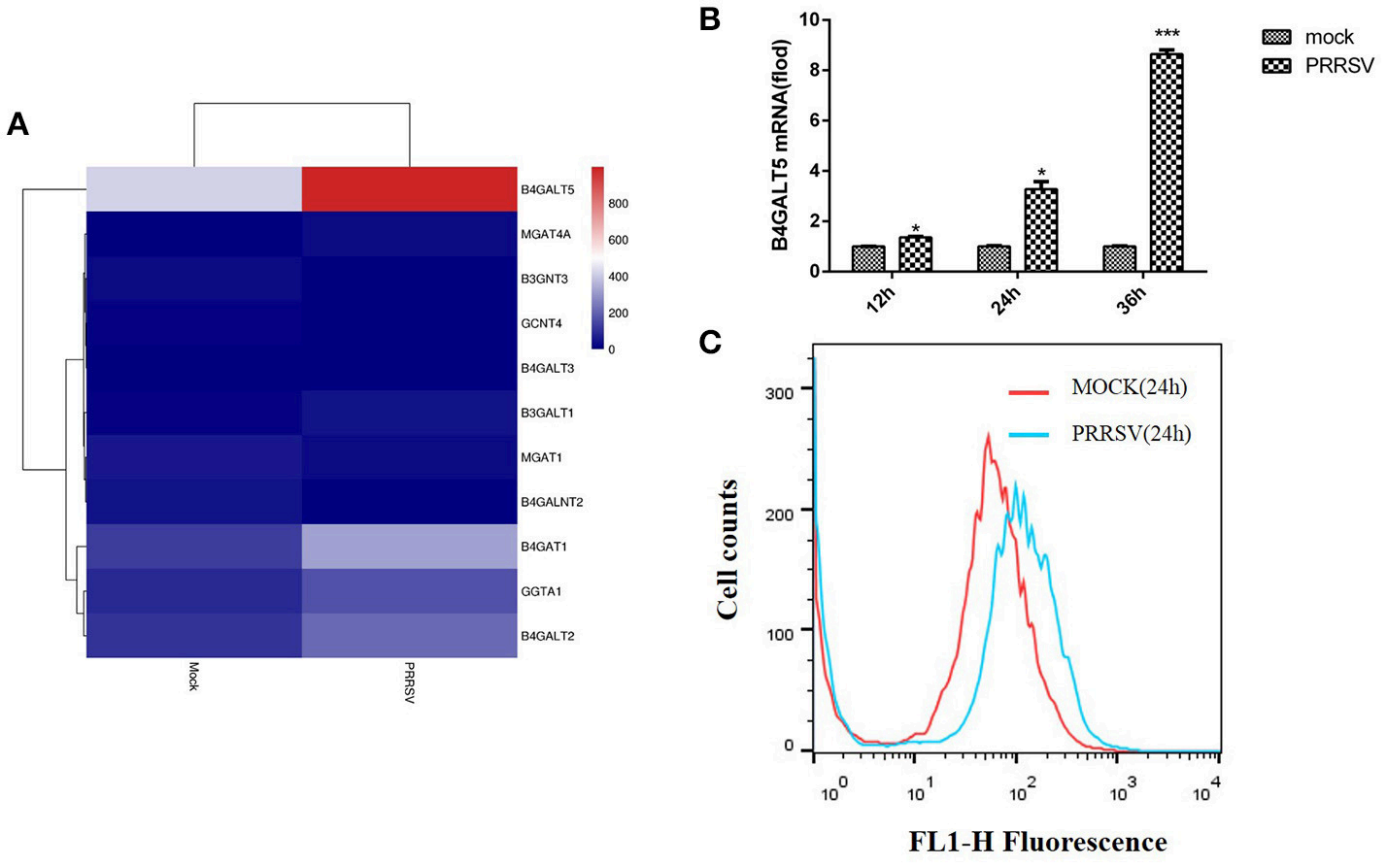
Porcine B4GALT5 expression was up-regulated during PRRSV infection. **(A)** The heatmap of glycosyltransferase-related differentially expressed genes. **(B)** 3D4/21 cells were mock-infected or infected with PRRSV at a multiplicity of infection (MOI) of 0.5. Cells were collected at the indicated time points, and subjected to real-time RT-PCR to analyze the expression of porcine B4GALT5. **(C)** 3D4/21 cells were mock-infected or infected with PRRSV at a multiplicity of infection (MOI) of 0.5. Cells were collected at the 24 h. Flow cytometry analysis of B4GALT5 protein expression levels. Differences in data were taken for be statistically significant if the *P*-value was less than 0.05 (^*^p < 0.05; ^***^p < 0.001).

A transcriptome analysis derived from the 3D4/21 cells was carried out for looking for related genes about signaling pathways and anti-viral immunity that had a significant change in the level of transcription after PRRSV infection, and also compared with other transcript data (Jiang et al., 2013; Islam et al., 2016). According to the data analysis, 3,815 genes were up-regulated after PRRSV infection and 2,435 genes were down-regulated (Supplementary Figure 1). B4GALT5 was found to be significantly upregulated among the glycosyltransferase genes after viral infection (Figure 1A). The results showed that the cellular B4GALT5 might have a certain relationship with the proliferation of the virus, which was selected for further study.

To verify whether PRRSV activates the expression of pB4GALT5, the mRNAs of pB4GALT5 from 3D4/21 cells infected with PRRSV of 0.5 MOI were detected by qRT-PCR. The transcription of pB4GALT5 was enhanced from 12 to 36 h than the untreated control (Figure 1B). The protein expression of pB4GALT5 was upregulated (Figure 1C). The above results testified that PRRSV upregulated pB4GALT5 expression in 3D4/21 cells.

### Domain and sequence analysis of porcine B4GALT5

Porcine B4GALT5 gene (complete CDS) was submitted to the GenBank (KY565579.1). And then, the gene encoded 388aa and existed transmembrane domains (Supplementary Figure 2). The pB4GALT5 protein included ligand binding sites, alpha helix, and beta strand.

**Figure 2:**
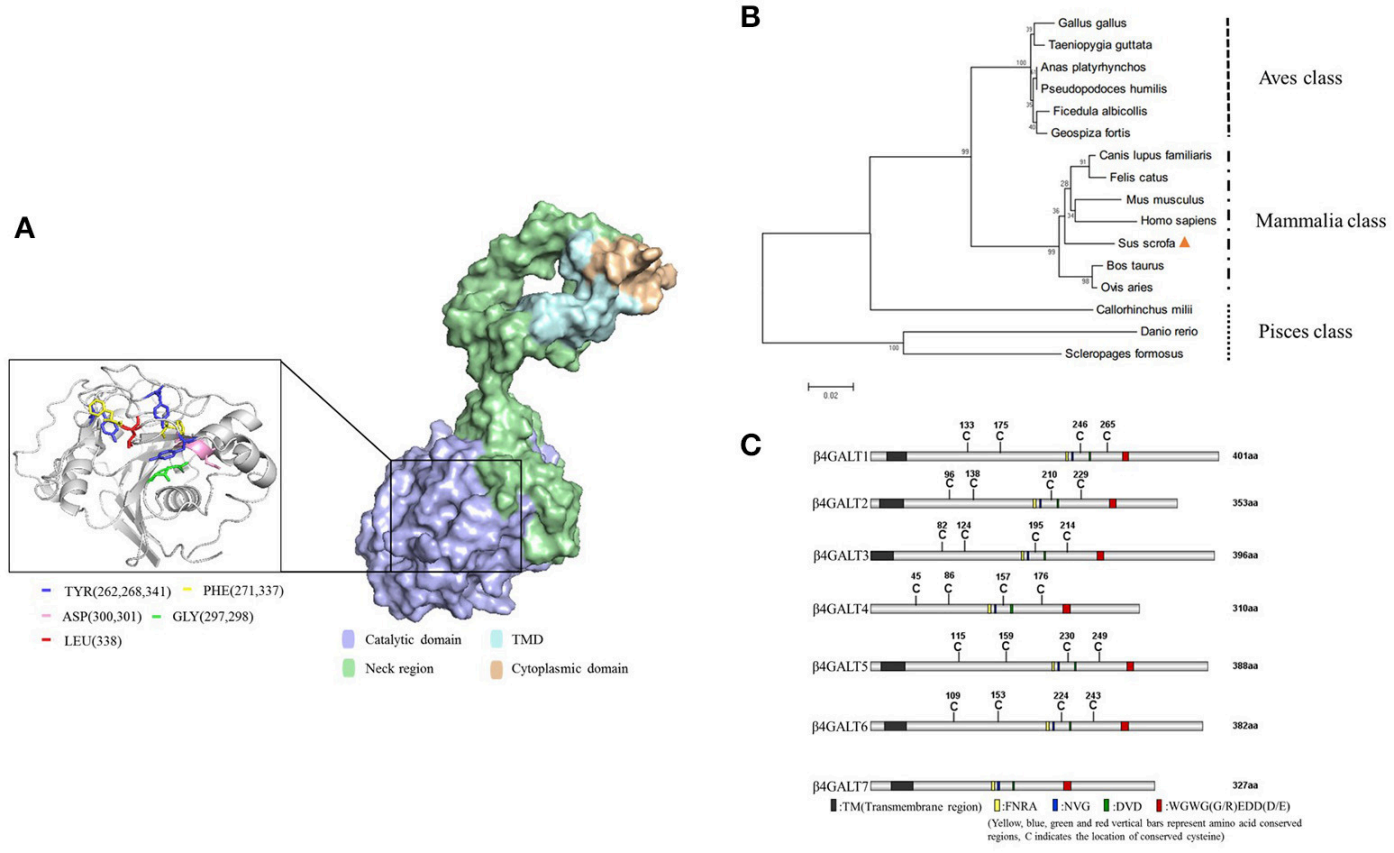
Domain and sequence analysis of porcine B4GALT5. **(A)** Protein 3D structure simulation using Zhang Lab's I-TASSER, PHYRE 2 online server and PyMOL software. The key amino acid sites that could bind ligand substrates were labeled (left) with different colors. **(B)** B4GALT5 phylogenetic tree of a range of species. Porcine B4GALT5 was indicated by a solid triangle, and all other B4GALT5 sequences were obtained from GeneBank. The unrooted phylogenetic tree was generated using neighbor-joining method by MEGA 5.0 software with 1,000 replications for bootstrap analysis and based on the alignment of B4GALT5 amino acid sequences. The scale bar is 0.02. **(C)** The amino acid sequence diagram of the family of porcine β-1, 4 galactosyltransferase, the homology was very low.

Like the other members of the B4GALT family, pB4GALT5 contained four domains: a cytoplasmic domain (1–12 aa of N-terminal) including abundant amino acids with positive charges; a transmembrane domain (TMD) (13–35 aa), which was rich in hydrophobic amino acids; a neck region (36–160 aa), which located in the Golgi apparatus lumen, containing glycine and proline residues; and a catalytic domain (161–388 aa), which was the largest and most important functional domain for pB4GALT5 in the lumen (Supplementary Figure 3A). Little was known with respect to the 3D structures of pB4GALT5. So, we modeled its protein 3D structure using Zhang Lab's I-TASSER. And the key part (161–388 aa) containing ligand binding sites was shown in Figure 2A.

The pB4GALT5 shared a high amino acid sequence similarity with homo sapiens B4GALT5 (hB4GALT5: NM_004776.3) and bos taurus B4GALT5 (bB4GALT5:XM_015466213.1) at 96.4 and 95.9%, respectively, but showed a relatively lower identity with danio rerio B4GALT5 (dViperin: NM_001045332.1) at 69.1% (Supplementary Figure 3B). The phylogenetic analysis revealed that pB4GALT5 might share a common ancestry with Homo sapiens and Mus muscluls. However, pB4GALT5 exhibited a relatively low identity to aves and pisces class (Figure 2B). To identify the differences in the amino acid sequence of the porcine B4GALT family, B4GALT1-7 amino acid sequences deriving from the NCBI database were aligned using DNAMAN software and the results were displayed using IBS (Illustrator for Biological Sequences) software (http://ibs.biocuckoo.org/). Obviously, B4GALT1 had the longest amino acid sequence and B4GALT7 had a short amino acid sequence. In addition to B4GALT7, they contained multiple conserved cysteine sites and four kind of conserved segments. Moreover, they were predicted to contain transmembrane domain except B4GALT4 (Figure 2C). Since the amino acid sequence of B4GALT4 was partial CDS, therefore, the analysis of the B4GALT4 only as a reference.

### Porcine B4GALT5 locates in the Golgi apparatus

It has been reported that human β-1, 4 galactosyltransferase belongs to type II membrane binding protein, mainly located in the Golgi apparatus (Rodeheffer and Shur, 2002). It is a single transmembrane protein, the amino-terminus is located in the cytoplasm, and the carboxyl-terminus is located in the Golgi lumen (Park et al., 2015).

**Figure 3:**
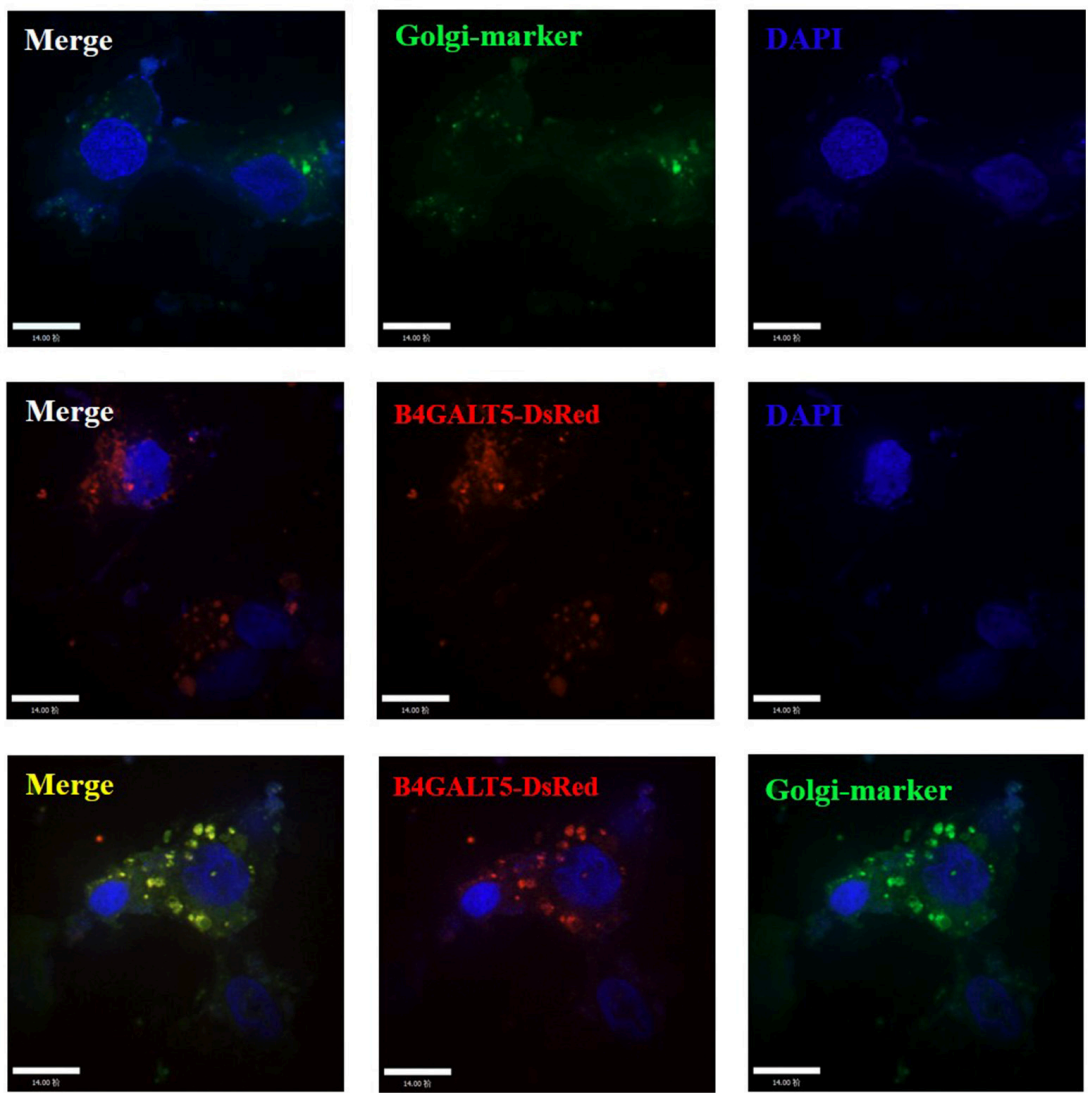
Subcellular localization analysis of porcine B4GALT5. 3D4/21 cells were transfected with GFP-Golgi marker and B4GALT5-DsRed. Cells were fixed and permeabilized at 24 h post-transfection. Cellular nuclei were stained with DAPI. The porcine B4GALT5 localization was observed under a laser confocal imaging analysis system, scale bar: 14 μm.

To investigate the location of porcine B4GALT5, we co-transfected the recombinant plasmids (GFP-Golgi marker protein and B4GALT5-DsRed) into 3D4/21 cells, the operation methods are listed in Materials and Methods. The results suggested that the fuse protein B4GALT5-DsRed had a co-located and gathered phenomenon with Golgi marker protein (Figure 3).

### The over-expression of B4GALT5 slightly inhibited the proliferation of PRRSV in 3D4/21 cells

**Figure 4:**
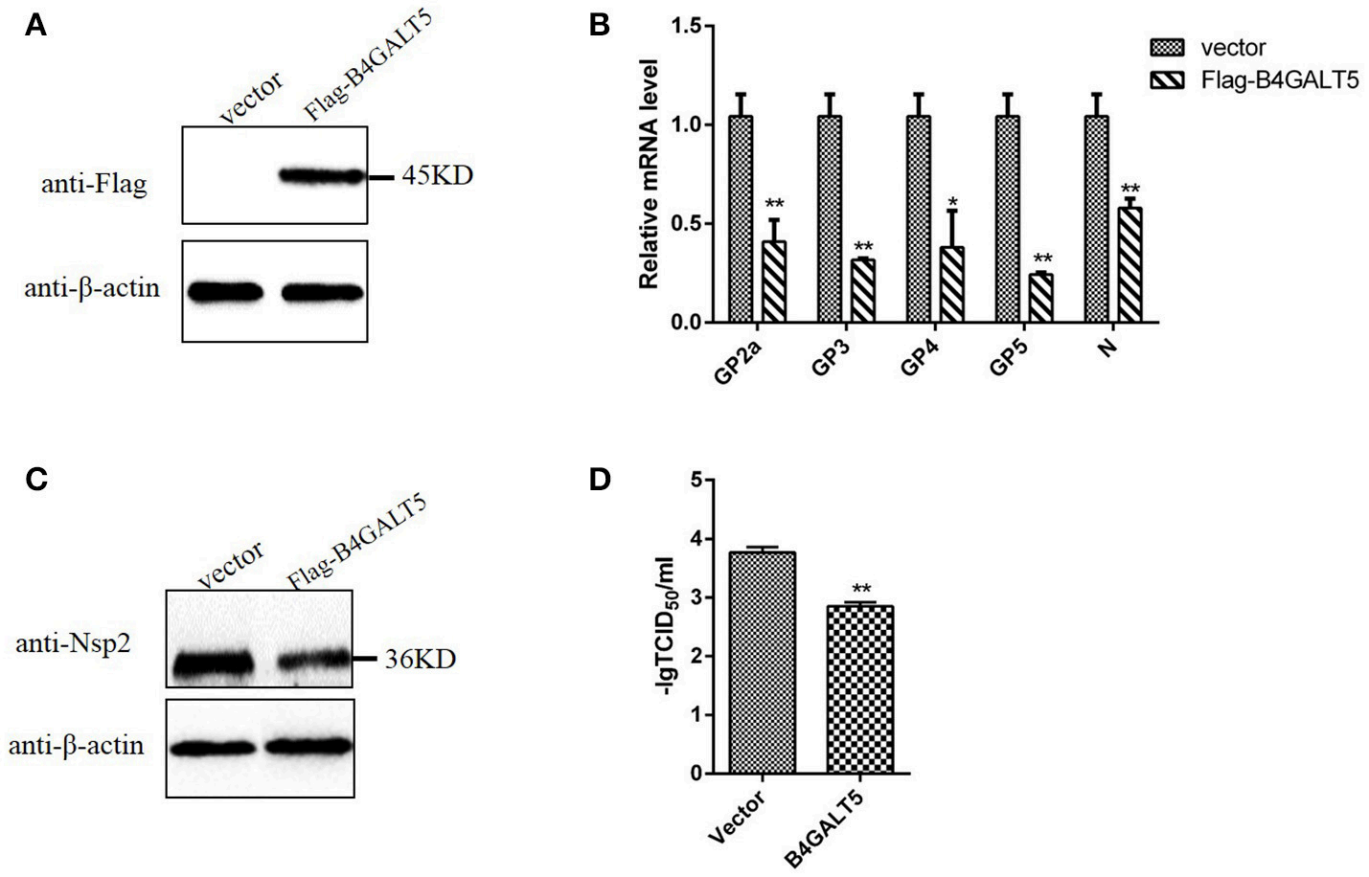
Inhibition of PRRSV replication by B4GALT5 over-expression in 3D4/21 cells. **(A)** The expression of Flag-B4GALT5 in 3D4/21 cells. 3D4/21 cells were transfected with Flag-B4GALT5 or empty vector as negative control. The Flag-B4GALT5 and empty vector were detected with a mouse anti-Flag mAb. β-actin was detected as an internal control using a mouse anti-β-actin mAb. **(B,C)** 3D4/21 cells were transfected with Flag-B4GALT5 or empty vector. Twenty-four hours after transfection, cells were infected with PRRSV (MOI 0.5). The infected cells were collected at 24 h post-infection, mRNA loads of viral structural genes (GP2a,GP3,GP4,GP5,N) were tested by qRT-PCR, and viral nonstructural protein (Nsp2) were tested by WB. **(D)** Next, PRRSV-containing samples were tested by TCID50 assay. All data represented the means and standard deviation of three independent experiments. Differences in data were taken for be statistically significant if the *P*-value was less than 0.05 (^*^p < 0.05; ^**^p < 0.01).

Firstly, western blot was implemented to examine Flag-B4GALT5 expression level in 3D4/21 cells transfected with Flag-B4GALT5 recombinant plasmid. As shown in Figure 4A, the expression of Flag-B4GALT5 could be observed. 3D4/21 cells that were expressing Flag-B4GALT5 were infected with PRRSV, the expression levels of virus protein and virus titers were analyzed after 24 h. As shown in Figure 4B, the transcription levels of virus envelope glycosylated-proteins (GP2a, 3, 4, 5) and structural protein (N) have different degrees of decline. Moreover, we also examined the viral non-structural protein (Nsp2) at protein levels, the results showed that Nsp2 protein expression could be inhibited slightly (Figure 4C). Furthermore, as shown in Figure 4D, the virus titers after the over-expression of pB4GALT5 were statistically down-regulated at 24 h after infection. All results indicated that the up-regulation of B4GALT5 inhibited the proliferation of PRRSV in 3D4/21 cells.

### Silencing of B4GALT5 improved the proliferation of PRRSV in 3D4/21 cells

**Figure 5:**
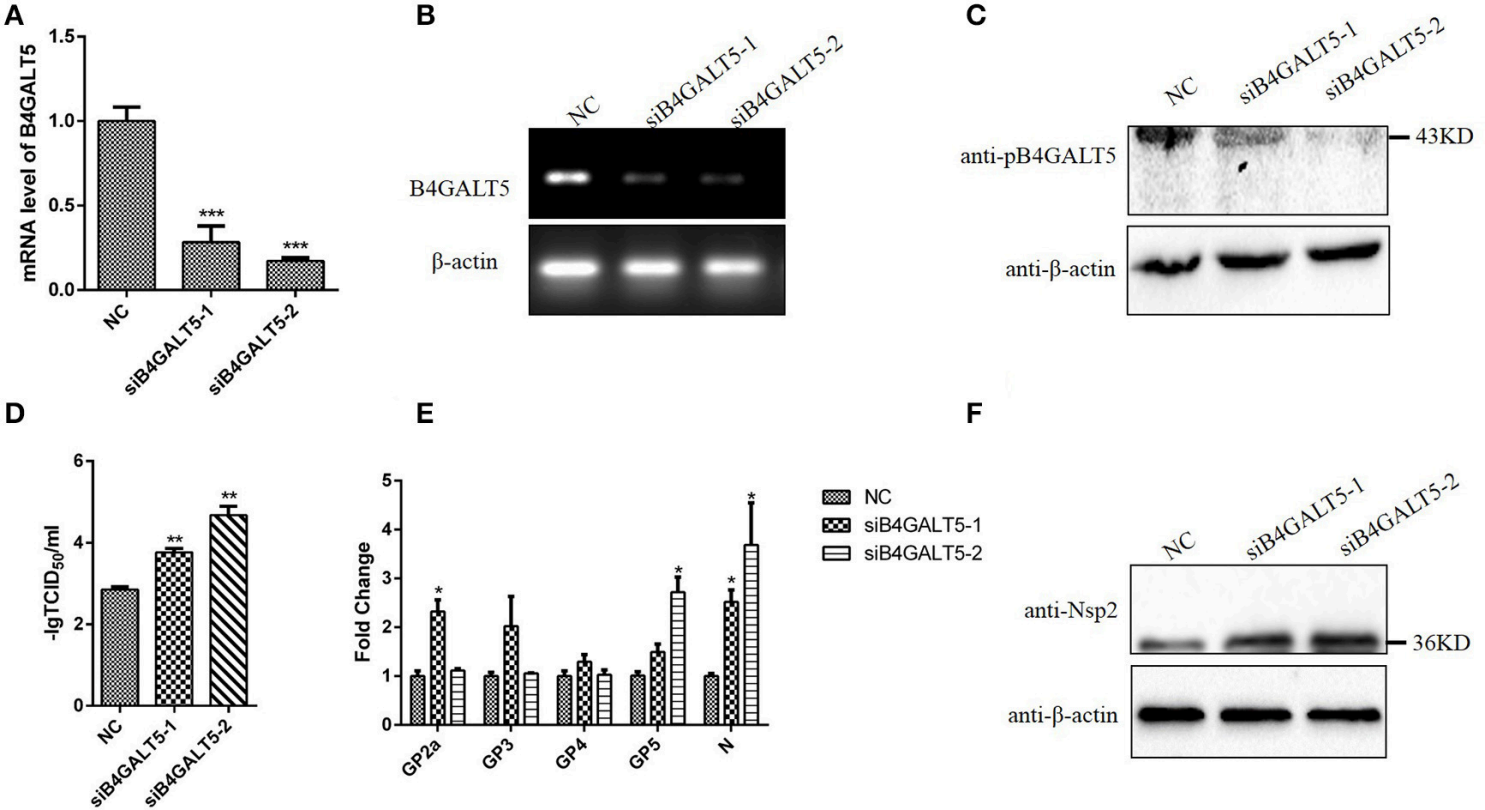
Silencing of B4GALT5 by siRNA improved the replication of PRRSV in 3D4/21 cells. **(A–C)** Silencing efficiency of B4GALT5 gene with three siRNAs (Sus-236, Sus-448 and NC) at the final concentration of 50 nmol. The expression level of B4GALT5 was examined at 48 h post-transfection by qRT-PCR and Western blot with a mouse anti-B4GALT5 mAb, β-actin was detected as an internal control using a mouse anti-β-actin mAb. **(D)** PRRSV titers in the supernatants of B4GALT5 gene-silenced 3D4/21 cells. 3D4/21 cells were transfected with Sus-236, Sus-448 and NC at a final concentration of 50 nmol, and were then infected at 24 h post-transfection with PRRSV at MOI of 0.5. The virus titers in the supernatants were assayed. **(E,F)** The PRRSV-infected and B4GALT5 gene-silenced 3D4/21 cells were collected at 24 h post-infection, mRNA loads of viral structural genes (GP2a,GP3,GP4,GP5,N) were tested by qRT-PCR, and viral nonstructural protein (Nsp2) were tested by Western Blot. Differences in data were taken for be statistically significant if the *P*-value was less than 0.05 (^*^p < 0.05; ^**^p < 0.01; ^***^p < 0.001).

Firstly, we verified the silence of pB4GALT5 in 3D4/21 cells by western blot and qRT-PCR after the cells were dealt with the final concentration of 50 nmol of two siRNAs (Sus-236 and Sus-448) targeting for B4GALT5 gene and negative control (NC) (Table 3). The results certified that the B4GALT5 expression could be restrained by two siRNAs (Figures 5A–C).

The 3D4/21 cells were incubated with PRRSV after transfected with the siRNA, and the mRNA and expression level of virus genes were assayed after infection. The results proved that mRNA and expression level of the siRNA-transfected cells were significantly high (Figures 5E,F). Additionally, the virus titers were statistically up-regulated (Figure 5D), indicating that silencing of B4GALT5 improved the proliferation of PRRSV in 3D4/21 cells.

### Porcine B4GALT5 interacted with the GP5 of PRRSV

**Figure 6:**
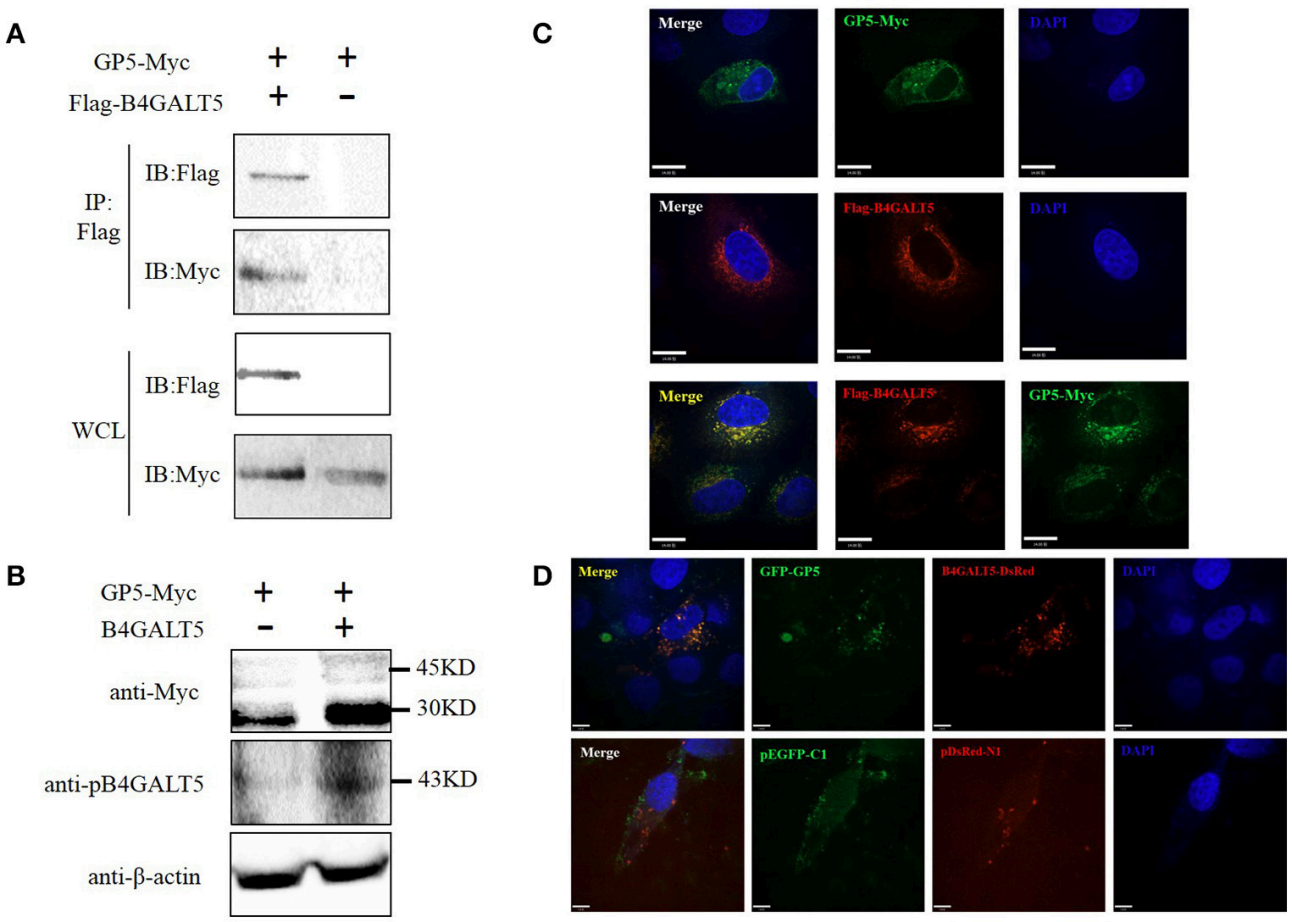
Identification of the interaction between pB4GALT5 and the GP5 of PRRSV. **(A)** Shown is the interaction of B4GALT5 with GP5 by Co-IP. 3D4/21 cells were co-transfected with the indicated plasmids. Thirty-six hours after transfection, the cell lysates were precipitated with an anti-Flag mAb in conjunction with protein A Sepharose, and further detected by Western blot with an anti-Flag mAb and an anti-Myc antibody, respectively. **(B)** Shown is the molecular weight of GP5-Myc increased and strip widened after the co-transfection of Flag-B4GALT5 and GP5-Myc by Western blot with an anti-Myc antibody. **(C)** Hela cells were co-transfected with GP5-Myc and Flag-B4GALT5. The cells at 36 h post-transfection were fixed and double-stained with a rabbit anti-Myc mAb and a mouse anti-Flag antibody, and followed by FITC-conjugated anti-rabbit IgG (green) and Cy5-conjugated anti-mouse IgG (red). Nuclei were stained with DAPI (blue). **(D)** 3D4/21 cells were co-transfected with GFP-GP5 and B4GALT5-DsRed. The cells at 36 h post-transfection were fixed and cellular nuclei were stained with DAPI. Above co-localizations were observed under a laser confocal imaging analysis system, scale bar: 14 μm.

Co-IP was carried out to verify the interaction between B4GALT5 and GP5. GP5-Myc and Flag-B4GALT5 were transfected to 3D4/21 cells. The Myc-tagged and Flag-tagged was detected with respective antibody by Western blot. The results showed that Flag-B4GALT5 could interact with GP5-Myc (Figure 6A). Additionally, the molecular weight of GP5-Myc increased and strip widened after the co-transfection of Flag-B4GALT5 and GP5-Myc (Figure 6B), indicating that GP5 was likely to be glycosylated by B4GALT5.

To determine whether B4GALT5 co-localized with GP5, GP5-Myc and Flag-B4GALT5 were transfected to Hela cells, and were detected at 36 h after transfection though laser confocal imaging analysis system. The photos declared that B4GALT5 and GP5 co-localized among the cytoplasm (Figure 6C). Moreover, the co-localization between GP5 and B4GALT5 was also observed in 3D4/21 cells co-transfected with GFP-GP5 and B4GALT5-DsRed (Figure 6D). In addition, we also explored the interaction between other GP and B4GALT5, but found no obvious co-localization phenomenon (Supplementary Figure 4).

### The over-expression of B4GALT5 facilitated the expression of immune-related molecules in 3D4/21 cells

**Figure 7:**
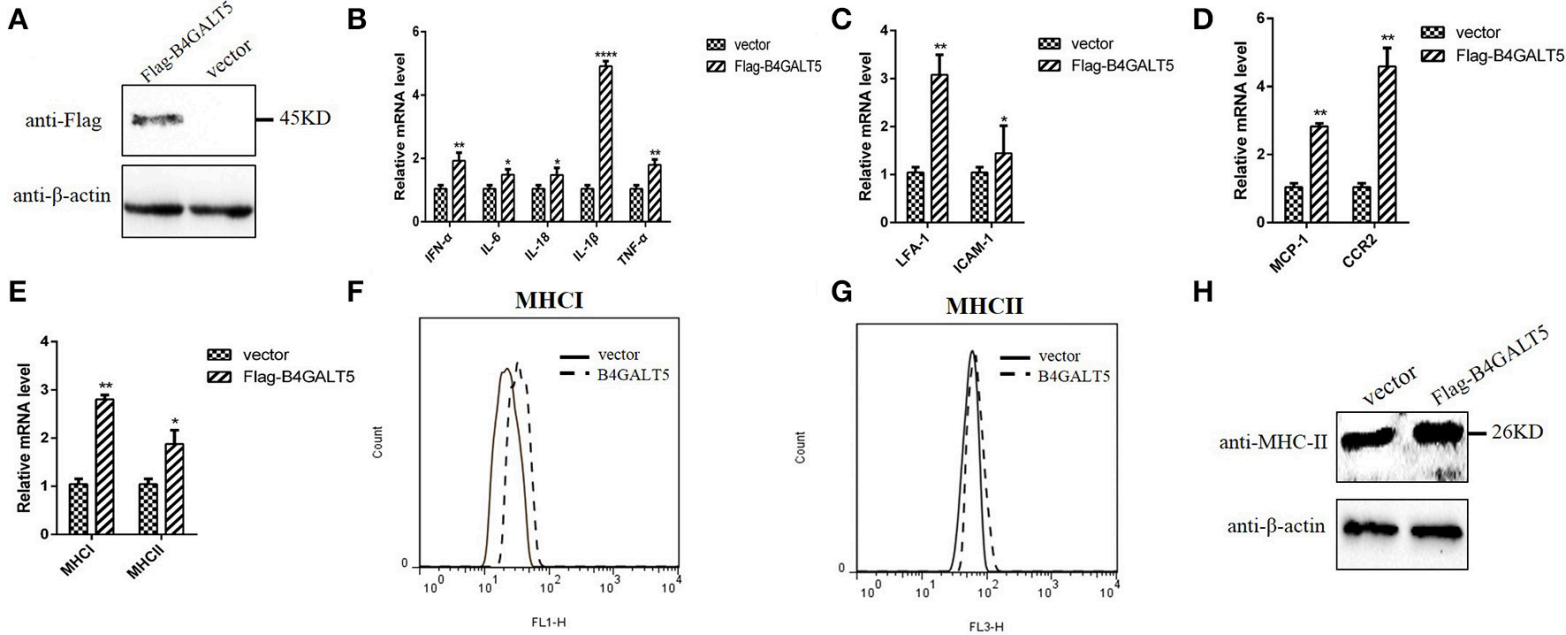
Facilitation of immune-related molecules expression by B4GALT5 over-expression in 3D4/21 cells. **(A)** The expression of Flag-B4GALT5 in 3D4/21 cells. 3D4/21 cells were transfected with Flag-B4GALT5 or empty vector as negative control. The Flag-B4GALT5 and empty vector were detected with a mouse anti-Flag mAb. β-actin was detected as an internal control using a mouse anti-β-actin mAb. **(B–E)** Analysis of transcription and expression of different cytokines produced by macrophages via qRT-PCR. **(F–H)** Protein expression levels of MHC-I, II by flow cytometry analysis and western blot. All data represented the means and standard deviation of three independent experiments. Differences in data were taken for be statistically significant if the *P*-value was less than 0.05 (^*^p < 0.05; ^**^p < 0.01; ^****^p < 0.0001).

3D4/21 cells over-expressed Flag-B4GALT5 (Figure 7A) that were then infected with the PRRSV at 0.5 MOI, the transcription of immune-related molecules produced by macrophages were upregulated with varying degrees by qRT-PCR, including interferon-α, interleukin (IL-6, 18) and other inflammatory factors (IL-1β, TNF-α), cell adhesion molecules (LFA-1, ICAM-1), chemokines (MCP-1, CCR2), and antigen presenting molecules (MHC-I, MHC-II) (Figures 7B–E).

The reasons for choosing these factors include the following two aspects. On the one hand, these immunomodulatory molecules were mainly produced in porcine macrophages (3D4/21) and involved in antigen presentation and anti-viral immunity. The B4GALT family involved in the inflammatory response, so inflammatory factors were also selected as the test subjects. On the other hand, they were cytokines or inflammatory factors that contain glycosylation sites. B4GALT5 encoded glycosyltransferase, we would like to explore whether B4GALT5 had an impact on the transcription and expression of glycoprotein-like immunomodulatory molecules.

Moreover, the glycosylated-protein expression of MHC-I, II were also promoted by over-expression of pB4GALT5 in 3D4/21 cells by flow cytometry assay and western blot (Figures 7F–H). Above results indicated that the over-expression of B4GALT5 facilitated the expression of glycosylated- immune molecules on cell surface in order to better resist the invasion of the virus.

### Silencing of B4GALT5 by siRNA suppressed the expression of immune-related molecules in 3D4/21 cells

**Figure 8:**
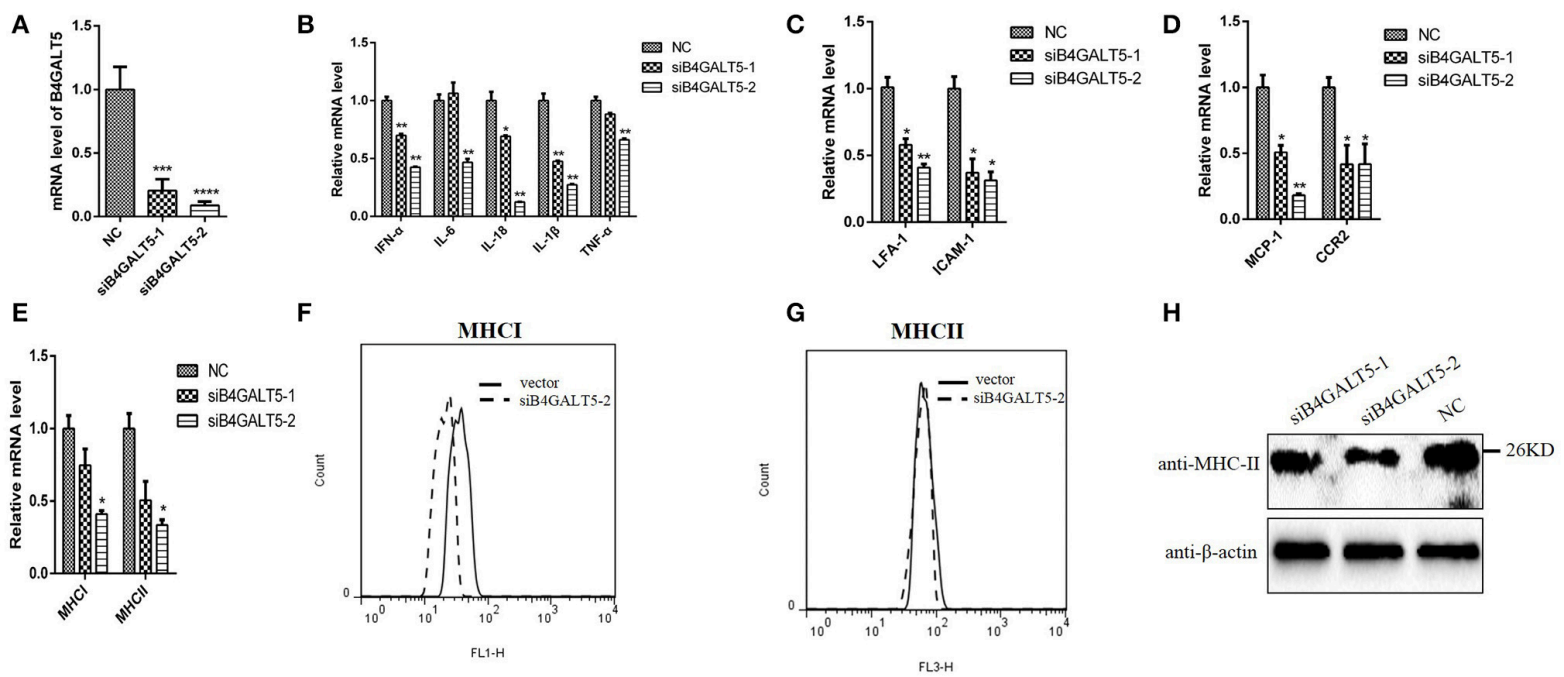
Silencing of B4GALT5 by siRNA suppressed immune-related molecules expression in 3D4/21 cells. **(A)** Silencing efficiency of B4GALT5 gene with three siRNAs (Sus-236, Sus-448 and NC) at the final concentration of 50 nmol. The expression level of B4GALT5 was examined at 48 h post-transfection by qRT-PCR. **(B–E)** The PRRSV-infected and B4GALT5 gene-silenced 3D4/21 cells were collected at 24 h post-infection, mRNA loads of immune-related molecules produced by macrophages were tested by qRT-PCR, **(F–H)** and antigen presenting molecules (MHC-I, MHC-II) were tested by flow cytometry analysis and western blot. Differences in data were taken for be statistically significant if the *P*-value was less than 0.05 (^*^p < 0.05; ^**^p < 0.01; ^***^p < 0.001; ^****^p < 0.0001).

3D4/21 cells that were treated with the final concentration of 50 nmol of two siRNAs (Sus-236 and Sus-448) targeting for B4GALT5 gene and negative control (NC) (Figure 8A), and then were incubated with PRRSV at 0.5 MOI, the transcription of immune-related molecules produced by macrophages were down-regulated with varying degrees by qRT-PCR, including interferon-α, interleukin (IL-6,18) and other inflammatory factors (IL-1β,TNF-α), cell adhesion molecules (LFA-1,ICAM-1), chemokines (MCP-1,CCR2), and antigen presenting molecules (MHC-I, MHC-II) (Figures 8B–E). Moreover, the glycosylated-protein expression of MHC-I, II were also suppressed in 3D4/21 cells by flow cytometry assay and western blot (Figures 8F–H), indicating that the silencing of B4GALT5 may affect the expression of immune-related factors thereby inhibiting their antiviral effects.

## Discussion

The glycosyltransferase was present in the post-translational modification of the protein, and the virus invaded the host cells and used the ribosomes, endoplasmic reticulum and Golgi in the host cells to translate and modify the self-structural proteins (Mardassi et al., 1996; Goodwin et al., 2015). β-1, 4 galactosyltransferase V carries out modification of important protein sugar chain in the Golgi through the synthesis of β-1,4 glycosidic bonds (Evans et al., 1995). β4GalT5 regulated the expression of Lewis-Y polysaccharides that played an important role in the embryo implantation process (Gu et al., 2015). Deletion of β4Ga1T5 might cause inhibition of lactose ceramide synthesis to lead placental developmental defects in mouse (Kumagai et al., 2009; Nishie et al., 2010). In mature 3T3-L1 adipocytes, TNF-α-induced insulin resistance was accompanied by a variety of changes in the sugar chain structure, including the increase in the expression of β4GalT5, suggesting that β4GalT5 might also be involved in the inflammatory response process (Parker et al., 2016). Overexpression of β4Ga1T5 could promote the invasion and survival of glioma cells, while inhibition of expression could promote apoptosis, inhibition of migration and invasion and tumor formation in nude mice (Jiang and Gu, 2010). Up to now, most studies about B4GALT5 concentrated mainly on human, rat, and mouse (Al-Obaide et al., 2015). However, no identified reports related to pig, *Sus scrofa*, was available. We firstly acquired complete CDS of pB4GALT5 and predicted structures. We also made the domain analyses and alignment and, indicating that pB4GALT5 shared a high identity with *Homo sapiens* and included a cytoplasmic domain, a neck region, a transmembrane domain and C-terminally catalytic domain. These findings laid the foundation for the study of its function.

The glycoproteins of the enveloped viruses and the glycoproteins in the host cell could utilize the same glycosyltransferases during protein glycosylation. To explore the relationship between β4Galt5 and the proliferation of enveloped viruses, we detected the transcription and protein levels of B4GALT5 in PRRSV-infected 3D4/21 cells. As shown as Figures 1B,C, the result indicated that PRRSV infection up-regulated pB4GALT5 expression in 3D4/21 cells. Here we proposed the hypothesis that β4Galt5 might be involved in the regulation of viral proliferation in 3D4/21 cells.

It had been reported that the envelope protein of PRRSV was rich in glycosylation sites (Ansari et al., 2006). The amino terminus of the GP5 protein extracellular domain was highly glycosylated in mature virus (Indik et al., 2000) and formed a heterologous dimer with M protein to constitute the main part of the viral capsule and complete the packaging of the virus (Wissink et al., 2005). Moreover, GP5 was a key protein for PRRSV invaded cells, and its glycosylation modification for viral infection was essential (Jiang et al., 2007). In this experiment, we tried to explore whether B4GALT5 would modify the GP5 and played an antiviral effect. The experimental results (Figure 6) show that B4GALT5 interacted with GP5, and it was reported that the steric effect of glycans would have an influence on the conformation of proteins and the formation of multimers (Bager et al., 2013). As shown as Figure 3, B4GALT5 located in Golgi lumen. Therefore, it was bold speculated that a number of GP5 might be trapped in Golgi lumen because of the interaction of GP5 and B4GALT5. GP5 was unable to participate in the assembly of the virus, so that complete virus particles could not be formed to inhibit the proliferation of the virus. Of course, the above speculations also required a lot of experiments to verify, we hope that following experiments can confirm the above conjectures, to provide new directions and targets for the development of anti-PRRSV infection drugs.

Moreover, the change of transcription and expression level of glycosyltransferase in enveloped virus infection cells may alter the glocoprotein modification on cell membrane, especially, the immune regulation prorein, such as the antigen presenting molecules, immune adhesion molecules and others to regulate the immune response. Macrophages as a class of cells with phagocytosis and antigen presentation were of great significance in the anti-viral immunity (Xie et al., 2017). Macrophages could initiate and enhance immune responses through the following two pathways and mediate inflammatory responses (Gajanayaka et al., 2017). Firstly, the role of antigen presentation to start immune response; Secondly, the secretion of various types of bioactive substances with immune enhancement effects (Voicu et al., 1994; Auray et al., 2016; Yu et al., 2016), including interferon (IFN-α), interleukin (IL-1β, IL-6, IL-18), chemokines (MCP-1) and receptor (CCR2), adhesion molecules (LFA-1, ICAM-1) (Liu et al., 2015), tumor necrosis factor (TNF-α) and antigen presenting molecules (MHC I, II). They could activate the biological function and promote the migration of macrophages. It was reported that all of the above important molecules involved in innate immunity were almost glycoprotein molecules (Krapp et al., 2003). In experimental results suggested that the transcription levels of cytokines secreted by macrophage cells had different degrees of upregulation in 3D4/21 cells over-expressed Flag-B4GALT5 that were then infected with the PRRSV at 0.5 MOI (Figures 7B–E). Therefore, it was speculated that, in the process of viral infection, B4GALT5 up-regulated the expression of antigen presenting molecules (MHC I, II) and adhesion molecules (LFA-1, ICAM-1) to strengthen the monitoring and presenting of the virus. Meanwhile, B4GALT5 up-regulated the expression of IFN-α and inflammatory factors (IL6, IL-18, IL-1β, TNF-α) to resist viral infection. In addition, B4GALT5 up-regulated the expression of chemokines (MCP-1) and receptor (CCR2), which attract more cells to migrate to the site of infection. They could supervise of pathogen intrusion. In addition, recent studies had shown that polysaccharides had immunomodulatory effects and would enhance non-specific immune function to improve macrophage phagocytosis (Li et al., 2017), however, β-1,4 galactosyltransferase might be involved in the synthesis of polysaccharides. The above speculations also need to be verified by the results of subsequent experiments.

In general, studies on β-1, 4 galactosyltransferases will promote the research progress of glycosylation of proteins. However, difficulties are also obvious. The reason is that the process of glycosylation modification is dynamic (Suryavathi et al., 2015; Moratz et al., 2017), and the connection of glycans is varied. At present, the researches about β-1, 4 galactosyltransferase mainly concentrate in β4GalT1 (Vanhooren et al., 2013; Sun et al., 2014), especially apoptosis-related regulatory mechanisms (Yuan et al., 2012). The studies of β4GalT2-7 remain to be strengthened. With the development of science, we hope that the important function of β-1, 4 galactosyltransferase will be explored in the fields of inflammatory reaction and immune regulation.

## Conclusion

Porcine B4GALT5 gene, a member of β-1, 4 galactosyltransferase (B4GALT) family, was acquired from porcine alveolar macrophages and located on the Golgi apparatus. The secondary and 3D structure modeling, domain analyses and alignment of amino acid sequence and performed in our study would make achievements by supporting the research of potent mechanisms of β-1, 4 galactosyltransferase V in antiviral immunity. Moreover, the expression of pB4GALT5 would be upregulated by PRRSV infection in 3D4/21 cells, down-regulated the replication of PRRSV by co-located and interaction with the GP5 of PRRSV, which provided new directions and targets for the development of anti-PRRSV infection drugs.

## Author contributions

JH: Conceived and designed the experiments; JR, PS, DL, YS, CZ, and LiZ: Performed the experiments; LeZ and JR: Analyzed the data; JH: Contributed reagents, materials, analysis tools; JR and JH: Wrote the paper.

### Conflict of interest statement

The authors declare that the research was conducted in the absence of any commercial or financial relationships that could be construed as a potential conflict of interest.

## References

[B1] Al-Ghouleh A. Johal R. Sharquie I. K. Emara M. Harrington H. Shakib F.. (2012). The glycosylation pattern of common allergens: the recognition and uptake of Der p 1 by epithelial and dendritic cells is carbohydrate dependent. PLoS ONE 7:e33929. 10.1371/journal.pone.0033929 22479478 PMC3316510

[B2] Al-Obaide M. A. Alobydi H. Abdelsalam A. G. Zhang R. Srivenugopal K. S. (2015). Multifaceted roles of 5'-regulatory region of the cancer associated gene B4GALT1 and its comparison with the gene family. Int. J. Oncol. 47, 1393–1404. 10.3892/ijo.2015.3136 26315939

[B3] Ansari I. H. Kwon B. Osorio F. A. Pattnaik A. K. (2006). Influence of N-linked glycosylation of porcine reproductive and respiratory syndrome virus GP5 on virus infectivity, antigenicity, and ability to induce neutralizing antibodies. J. Virol. 80, 3994–4004. 10.1128/JVI.80.8.3994-4004.2006 16571816 PMC1440468

[B4] Auray G. Lachance C. Wang Y. Gagnon C. A. Segura M. Gottschalk M. (2016). Transcriptional analysis of PRRSV-infected porcine dendritic cell response to streptococcus suis infection reveals up-regulation of inflammatory-related genes expression. PLoS ONE 11:e0156019. 10.1371/journal.pone.0156019 27213692 PMC4877111

[B5] Bagdonaite I. Nordén R. Joshi H. J. King S. L. Vakhrushev S. Y. Olofsson S.. (2016). Global mapping of O-glycosylation of varicella zoster virus, human cytomegalovirus, and epstein-barr virus. J. Biol. Chem. 291, 12014–12028. 10.1074/jbc.M116.721746 27129252 PMC4933254

[B6] Bager R. Johansen J. S. Jensen J. K. Stensballe A. Jendroszek A. Buxbom L.. (2013). Protein conformational change delayed by steric hindrance from an N-linked glycan. J. Mol. Biol. 425, 2867–2877. 10.1016/j.jmb.2013.05.007 23702291

[B7] Bautista E. M. Meulenberg J. J. Choi C. S. Molitor T. W. (1996). Structural polypeptides of the American (VR-). strain of porcine reproductive and respiratory syndrome virus. Arch. Virol. 141, 1357–1365. 10.1007/BF01718837 8774694

[B8] Cartault F. Munier P. Jacquemont M. L. Vellayoudom J. Doray B. Payet C.. (2015). Expanding the clinical spectrum of B4GALT7 deficiency: homozygous p.R270C mutation with founder effect causes Larsen of Reunion Island syndrome. Eur. J. Hum. Genet. 23, 49–53. 10.1038/ejhg.2014.60 24755949 PMC4266744

[B9] Conzelmann K. K. Visser N. Van Woensel P. Thiel H. J. (1993). Molecular characterization of porcine reproductive and respiratory syndrome virus, a member of the arterivirus group. Virology 193, 329–339. 10.1006/viro.1993.1129 8438574 PMC7131490

[B10] DeVito S. R. Ortiz-Riaño E. Martínez-Sobrido L. Munger J. (2014). Cytomegalovirus-mediated activation of pyrimidine biosynthesis drives UDP-sugar synthesis to support viral protein glycosylation. Proc. Natl. Acad. Sci. U.S.A. 111, 18019–18024. 10.1073/pnas.1415864111 25472841 PMC4273352

[B11] Evans S. C. Youakim A. Shur B. D. (1995). Biological consequences of targeting beta 1,4-galactosyltransferase to two different subcellular compartments. BioEssays 17, 261–268. 10.1002/bies.950170313 7748180

[B12] Fan Y. Yu L. Zhang Q. Jiang Y. Dai F. Chen C.. (1999). Cloning and characterization of a novel member of human beta-1,4-galactosyltransferase gene family. Sci. China C Life Sci. 42, 337–345. 10.1007/BF02882052 18763123

[B13] Furukawa K. (2015). Challenge to the suppression of tumor growth by the beta4-galactosyltransferase genes. Proc. Jpn. Acad. B. Phys. Biol. Sci. 91, 1–16. 10.2183/pjab.91.1 25743061 PMC4405391

[B14] Gajanayaka N. O'Hara S. Konarski Y. Fernandes J. Muthumani K. Kozlowski M.. (2017). HIV and HIV-Tat inhibit LPS-induced IL-27 production in human macrophages by distinct intracellular signaling pathways. J. Leukoc. Biol. 102, 925–939. 10.1189/jlb.4A0716-332RR 28698313

[B15] Gao J. Ji P. Zhang M. Wang X. Li N. Wang C.. (2014). GP5 expression in Marc-145 cells inhibits porcine reproductive and respiratory syndrome virus infection by inducing beta interferon activity. Vet. Microbiol. 174, 409–418. 10.1016/j.vetmic.2014.09.030 25457367

[B16] Goodwin C. M. Xu S. Munger J. (2015). Stealing the keys to the kitchen: viral manipulation of the host cell metabolic network. Trends Microbiol. 23, 789–798. 10.1016/j.tim.2015.08.007 26439298 PMC4679435

[B17] Gu J. Fan J. Xu Y. Xie Y. Gong T. Kong Y. (2015). Regulatory function of beta1,4-galactosyltransferase I expression on Lewis-Y glycan and embryo implantation. Gene 562, 220–225. 10.1016/j.gene.2015.02.072 25735572

[B18] Indik S. Valícek L. Klein D. Klánová J. (2000). Variations in the major envelope glycoprotein GP5 of Czech strains of porcine reproductive and respiratory syndrome virus. J. Gen. Virol. 81, 2497–2502. 10.1099/0022-1317-81-10-2497 10993939

[B19] Islam M. A. Große-Brinkhaus C. Pröll M. J. Uddin M. J. Rony S. A. Tesfaye D.. (2016). Deciphering transcriptome profiles of peripheral blood mononuclear cells in response to PRRSV vaccination in pigs. BMC Genomics 17:641. 10.1186/s12864-016-2849-1 27528396 PMC4986384

[B20] Jiang J. Gu J. (2010). Beta1,4-galactosyltransferase V A growth regulator in glioma. Meth. Enzymol. 479, 3–23. 10.1016/S0076-6879(10)79001-7 20816157

[B21] Jiang W. Jiang P. Wang X. Li Y. Wang X. Du Y. (2007). Influence of porcine reproductive and respiratory syndrome virus GP5 glycoprotein N-linked glycans on immune responses in mice. Virus Genes 35, 663–671. 10.1007/s11262-007-0131-y 17671839

[B22] Jiang Z. Zhou X. Michal J. J. Wu X. L. Zhang L. Zhang M.. (2013). Reactomes of porcine alveolar macrophages infected with porcine reproductive and respiratory syndrome virus. PLoS ONE 8:e59229. 10.1371/journal.pone.0059229 23527143 PMC3602036

[B23] Khan A. A. Bose C. Yam L. S. Soloski M. J. Rupp F. (2001). Physiological regulation of the immunological synapse by agrin. Science 292, 1681–1686. 10.1126/science.1056594 11349136

[B24] Krapp S. Mimura Y. Jefferis R. Huber R. Sondermann P. (2003). Structural analysis of human IgG-Fc glycoforms reveals a correlation between glycosylation and structural integrity. J. Mol. Biol. 325, 979–989. 10.1016/S0022-2836(02)01250-0 12527303

[B25] Kumagai T. Tanaka M. Yokoyama M. Sato T. Shinkai T. Furukawa K. (2009). Early lethality of beta-1,4-galactosyltransferase V-mutant mice by growth retardation. Biochem. Biophys. Res. Commun. 379, 456–459. 10.1016/j.bbrc.2008.12.078 19114028

[B26] Li M. Yan Y. X. Yu Q. T. Deng Y. Wu D. T. Wang Y.. (2017). Comparison of immunomodulatory effects of fresh garlic and black garlic polysaccharides on RAW 264.7 Macrophages. J. Food Sci. 82, 765–771. 10.1111/1750-3841.13589 28196294

[B27] Liu J. Hou M. Yan M. Lü X. Gu W. Zhang S.. (2015). ICAM-1-dependent and ICAM-1-independent neutrophil lung infiltration by porcine reproductive and respiratory syndrome virus infection. Am. J. Physiol. Lung Cell. Mol. Physiol. 309, L226–L236. 10.1152/ajplung.00037.2015 26001774

[B28] Mardassi H. Massie B. Dea S. (1996). Intracellular synthesis, processing, and transport of proteins encoded by ORFs 5 to 7 of porcine reproductive and respiratory syndrome virus. Virology 221, 98–112. 10.1006/viro.1996.0356 8661418

[B29] Moratz J. Klepel F. Ravoo B. J. (2017). Dynamic glycosylation of liposomes by thioester exchange. Org. Biomol. Chem. 15, 5089–5094. 10.1039/C7OB00805H 28585976

[B30] Nishie T. Hikimochi Y. Zama K. Fukusumi Y. Ito M. Yokoyama H.. (2010). Beta4-galactosyltransferase-5 is a lactosylceramide synthase essential for mouse extra-embryonic development. Glycobiology 20, 1311–1322. 10.1093/glycob/cwq098 20574042

[B31] Park J. H. Hogrebe M. Grüneberg M. DuChesne I. von der Heiden A. L. Reunert J.. (2015). SLC39A8 deficiency: a disorder of manganese transport and glycosylation. Am. J. Hum. Genet. 97, 894–903. 10.1016/j.ajhg.2015.11.003 26637979 PMC4678430

[B32] Parker B. L. Thaysen-Andersen M. Fazakerley D. J. Holliday M. Packer N. H. James D. E. (2016). Terminal galactosylation and sialylation switching on membrane glycoproteins upon TNF-alpha-induced insulin resistance in adipocytes. Mol. Cell. Proteomics 15, 141–153. 10.1074/mcp.M115.054221 26537798 PMC4762517

[B33] Rodeheffer C. Shur B. D. (2002). Targeted mutations in beta1,4-galactosyltransferase I reveal its multiple cellular functions. Biochim. Biophys. Acta 1573, 258–270. 10.1016/S0304-4165(02)00392-6 12417408

[B34] Schmid J. Heider D. Wendel N. J. Sperl N. Sieber V. (2016). Bacterial glycosyltransferases: challenges and opportunities of a highly diverse enzyme class toward tailoring natural products. Front. Microbiol. 7:182. 10.3389/fmicb.2016.00182 26925049 PMC4757703

[B35] Shen A. Yan J. Ding F. Gu X. Zhu D. Gu J. (2003a). Overexpression of beta-1,4-galactosyltransferase I in rat Schwann cells promotes the growth of co-cultured dorsal root ganglia. Neurosci. Lett. 342, 159–162. 10.1016/S0304-3940(03)00271-4 12757889

[B36] Shen A. Zhu D. Ding F. Zhu M. Gu X. Gu J. (2003b). Increased gene expression of beta-1,4-galactosyltransferase I in rat injured sciatic nerve. J. Mol. Neurosci. 21, 103–110. 10.1385/JMN:21:2:103 14593210

[B37] Shirane K. Kuji R. Tareyanagi C. Furukawa K. (2014). Gene expression levels of beta4-galactosyltransferase 5 correlate with the tumorigenic potentials of B16-F10 mouse melanoma cells. Glycobiology 24, 532–541. 10.1093/glycob/cwu021 24653215

[B38] Snijder E. J. van Tol H. Pedersen K. W. Raamsman M. J. de Vries A. A. (1999). Identification of a novel structural protein of arteriviruses. J. Virol. 73, 6335–6345. 10400725 10.1128/jvi.73.8.6335-6345.1999 PMC112712

[B39] Sun X. Wu Y. Wang Y. Xue Q. Cheng X. Zhang G.. (2014). beta-1,4-galactosyltransferase-I activates proliferation and participates in intercellular contacts of lymphocytes. Hum. Immunol. 75, 1019–1025. 10.1016/j.humimm.2014.08.199 25223470

[B40] Suryavathi V. Panneerdoss S. Wolkowicz M. J. Shetty J. Sherman N. E. Flickinger C. J.. (2015). Dynamic changes in equatorial segment protein 1 (SPESP1). Glycosylation during mouse spermiogenesis. Biol. Reprod. 92:129. 10.1095/biolreprod.114.121095 25761597 PMC4645982

[B41] Tavares-Carreón F. Fathy Mohamed Y. Andrade A. Valvano M. A. (2016). ArnT proteins that catalyze the glycosylation of lipopolysaccharide share common features with bacterial N-oligosaccharyltransferases. Glycobiology 26, 286–300. 10.1093/glycob/cwv095 26515403 PMC4736538

[B42] Terasaka K. Mizutani Y. Nagatsu A. Mizukami H. (2012). In situ UDP-glucose regeneration unravels diverse functions of plant secondary product glycosyltransferases. FEBS Lett. 586, 4344–4350. 10.1016/j.febslet.2012.10.045 23159939

[B43] Ujita M. Misra A. K. McAuliffe J. Hindsgaul O. Fukuda M. (2000). Poly-N-acetyllactosamine extension in N-glycans and core 2- and core 4-branched O-glycans is differentially controlled by i-extension enzyme and different members of the beta 1,4-galactosyltransferase gene family. J. Biol. Chem. 275, 15868–15875. 10.1074/jbc.M001034200 10747980

[B44] Vanhooren V. Vandenbroucke R. E. Dewaele S. Van Hamme E. Haigh J. J. Hochepied T.. (2013). Mice overexpressing beta-1,4-Galactosyltransferase I are resistant to TNF-induced inflammation and DSS-induced colitis. PLoS ONE 8:e79883. 10.1371/journal.pone.0079883 24339869 PMC3855152

[B45] Voicu I. L. Silim A. Morin M. Elazhary M. A. (1994). Interaction of porcine reproductive and respiratory syndrome virus with swine monocytes. Vet. Rec. 134, 422–423. 10.1136/vr.134.16.422 8036775

[B46] Wang D. Fang L. Zhao F. Luo R. Chen H. Xiao S. (2011). Molecular cloning, expression and antiviral activity of porcine interleukin-29 (poIL-29). Dev. Comp. Immunol. 35, 378–384. 10.1016/j.dci.2010.11.003 21078342

[B47] Wasik B. R. Barnard K. N. Parrish C. R. (2016). Effects of sialic acid modifications on virus binding and infection. Trends Microbiol. 24, 991–1001. 10.1016/j.tim.2016.07.005 27491885 PMC5123965

[B48] Wei Y. Zhou F. Ge Y. Chen H. Cui C. Li Q.. (2010). Beta1,4-galactosyltransferase V regulates self-renewal of glioma-initiating cell. Biochem. Biophys. Res. Commun. 396, 602–607. 10.1016/j.bbrc.2010.04.110 20417617

[B49] Wissink E. H. Kroese M. V. van Wijk H. A. Rijsewijk F. A. Meulenberg J. J. Rottier P. J. (2005). Envelope protein requirements for the assembly of infectious virions of porcine reproductive and respiratory syndrome virus. J. Virol. 79, 12495–12506. 10.1128/JVI.79.19.12495-12506.2005 16160177 PMC1211556

[B50] Xie D. L. Zheng M. M. Zheng Y. Gao H. Zhang J. Zhang T.. (2017). Vibrio vulnificus induces mTOR activation and inflammatory responses in macrophages. PLoS ONE 12:e0181454. 10.1371/journal.pone.0181454 28719654 PMC5515453

[B51] Yu Z. Huang C. Zhang Q. Feng W. H. (2016). Porcine reproductive and respiratory syndrome virus (PRRSV). induces IL-12p40 production through JNK-AP-1 and NF-kappaB signaling pathways. Virus Res. 225, 73–81. 10.1016/j.virusres.2016.09.009 27663131

[B52] Yuan Q. Yang H. Cheng C. Li C. Wu X. Huan W.. (2012). beta-1,4-Galactosyltransferase I involved in Schwann cells proliferation and apoptosis induced by tumor necrosis factor-alpha via the activation of MAP kinases signal pathways. Mol. Cell. Biochem. 365, 149–158. 10.1007/s11010-012-1254-6 22359038

[B53] Zhang H. Liu Y. Xie H. Fu Q. Liu Z. Zhu Y.. (2017). Beta-1,4-galactosyltransferase II predicts poor prognosis of patients with non-metastatic clear-cell renal cell carcinoma. Tumour Biol. 39:1010428317691417. 10.1177/1010428317691417 28231735

[B54] Zhou H. Ma H. Wei W. Ji D. Song X. Sun J.. (2013). B4GALT family mediates the multidrug resistance of human leukemia cells by regulating the hedgehog pathway and the expression of p-glycoprotein and multidrug resistance-associated protein 1. Cell Death Dis. 4:e654. 10.1038/cddis.2013.186 23744354 PMC3698553

