# Supplementary material for: The Immunological Regulation Roles of Porcine β-1, 4 Galactosyltransferase V (B4GALT5) in PRRSV Infection
Source: Front Cell Infect Microbiol. 2018 Mar 1;8:48. doi: 10.3389/fcimb.2018.00048 (PMC5837993; doi:10.3389/fcimb.2018.00048)
Supplement: Supplementary file 1 [file Presentation1.PPT]

## Slide 1
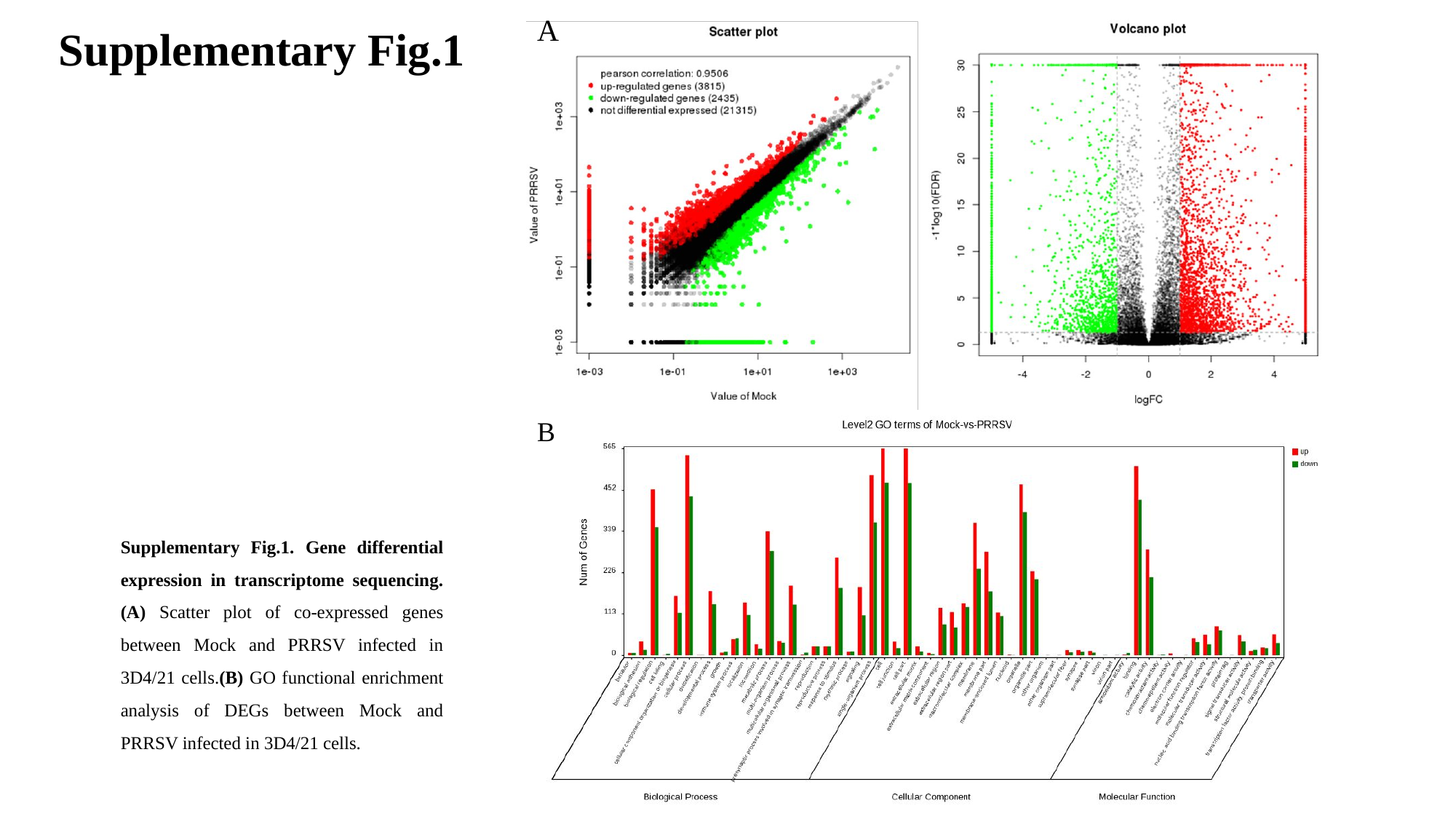

A
B
Supplementary Fig.1
Supplementary Fig.1. Gene differential expression in transcriptome sequencing. (A) Scatter plot of co-expressed genes between Mock and PRRSV infected in 3D4/21 cells.(B) GO functional enrichment analysis of DEGs between Mock and PRRSV infected in 3D4/21 cells.

## Slide 2
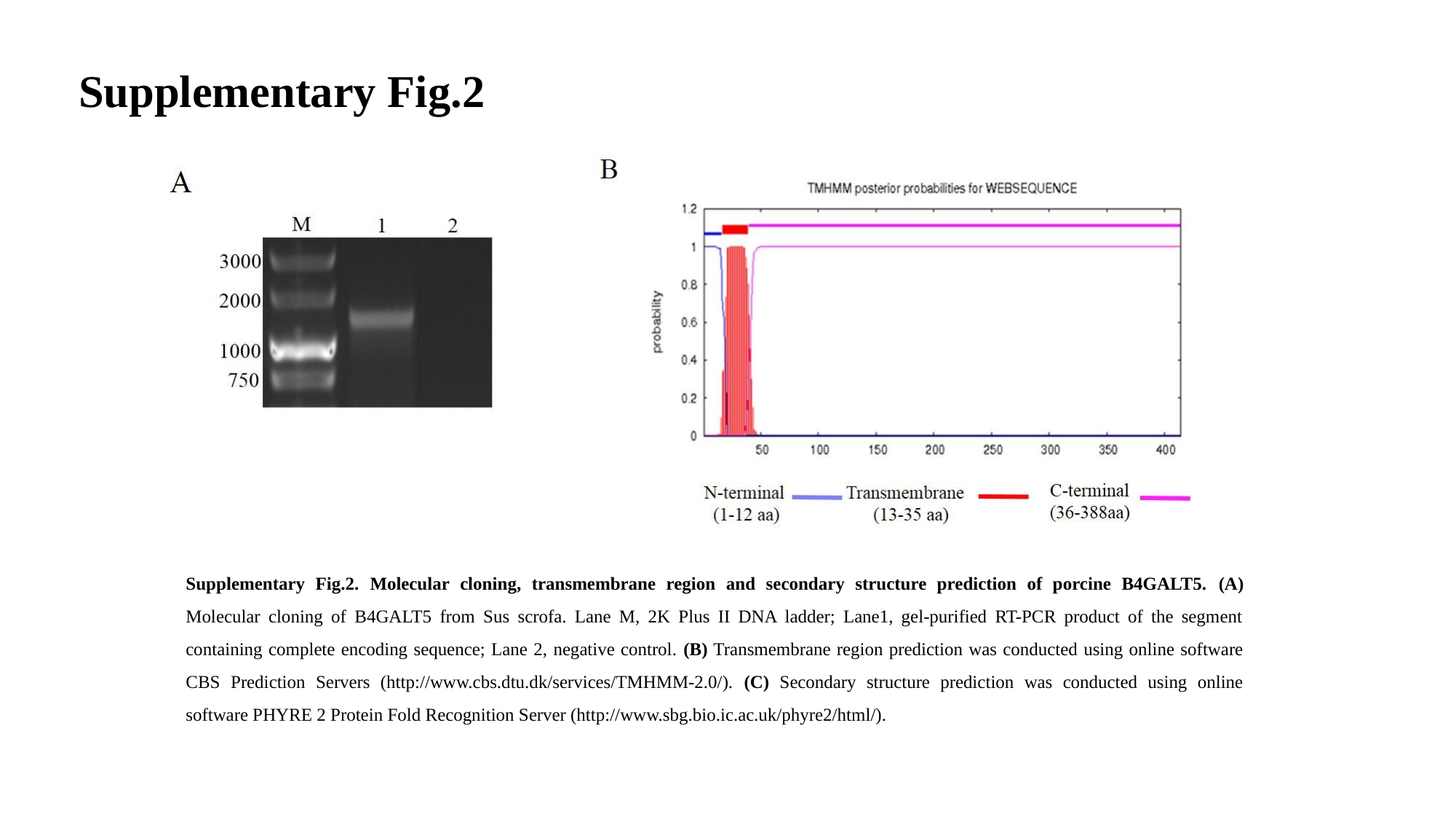

Supplementary Fig.2
Supplementary Fig.2. Molecular cloning, transmembrane region and secondary structure prediction of porcine B4GALT5. (A) Molecular cloning of B4GALT5 from Sus scrofa. Lane M, 2K Plus II DNA ladder; Lane1, gel-purified RT-PCR product of the segment containing complete encoding sequence; Lane 2, negative control. (B) Transmembrane region prediction was conducted using online software CBS Prediction Servers (http://www.cbs.dtu.dk/services/TMHMM-2.0/). (C) Secondary structure prediction was conducted using online software PHYRE 2 Protein Fold Recognition Server (http://www.sbg.bio.ic.ac.uk/phyre2/html/).

## Slide 3
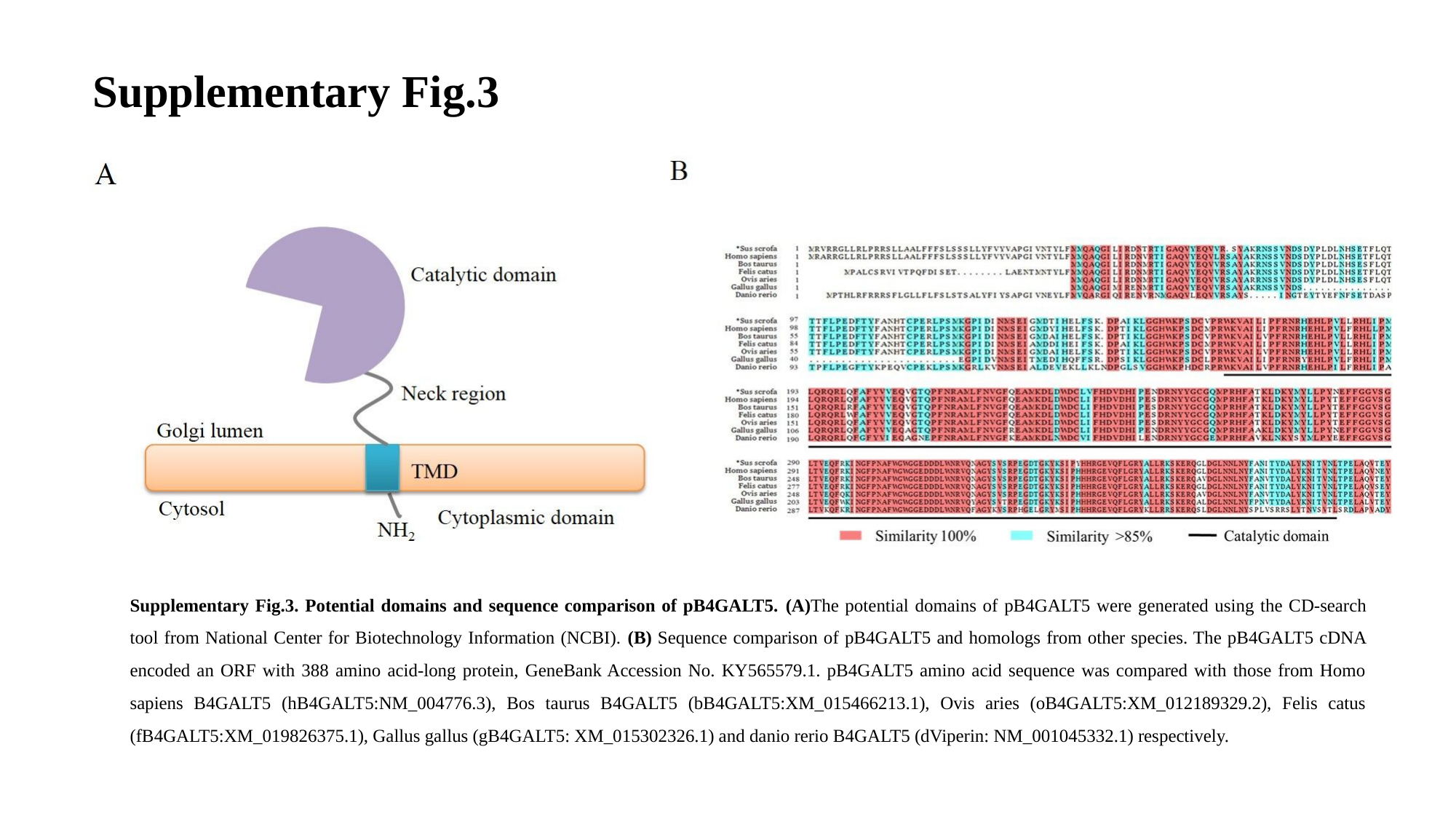

Supplementary Fig.3
Supplementary Fig.3. Potential domains and sequence comparison of pB4GALT5. (A)The potential domains of pB4GALT5 were generated using the CD-search tool from National Center for Biotechnology Information (NCBI). (B) Sequence comparison of pB4GALT5 and homologs from other species. The pB4GALT5 cDNA encoded an ORF with 388 amino acid-long protein, GeneBank Accession No. KY565579.1. pB4GALT5 amino acid sequence was compared with those from Homo sapiens B4GALT5 (hB4GALT5:NM_004776.3), Bos taurus B4GALT5 (bB4GALT5:XM_015466213.1), Ovis aries (oB4GALT5:XM_012189329.2), Felis catus (fB4GALT5:XM_019826375.1), Gallus gallus (gB4GALT5: XM_015302326.1) and danio rerio B4GALT5 (dViperin: NM_001045332.1) respectively.

## Slide 4
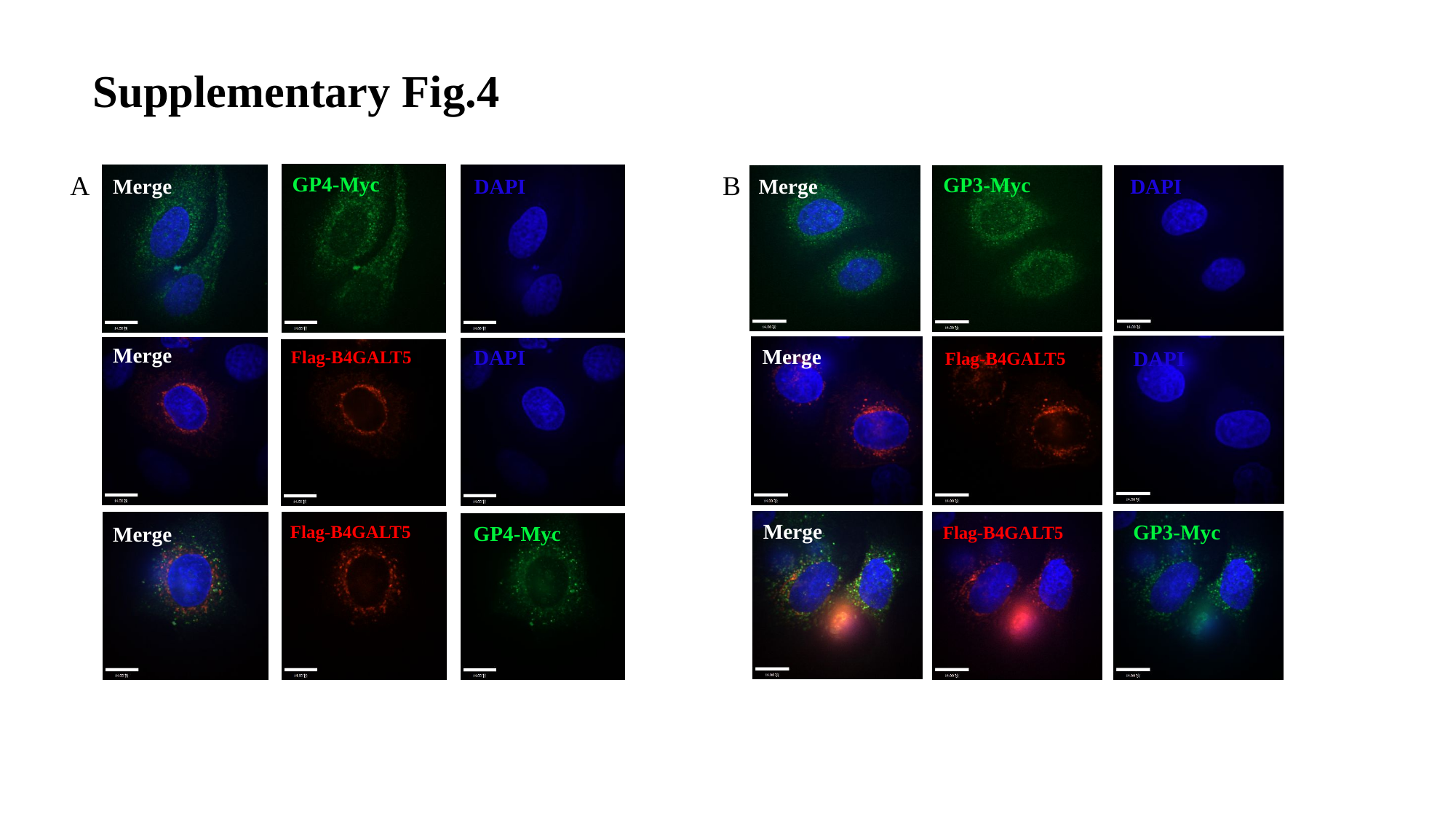

Supplementary Fig.4
A
GP4-Myc
DAPI
Merge
Merge
DAPI
Flag-B4GALT5
Flag-B4GALT5
GP4-Myc
Merge
B
GP3-Myc
DAPI
Merge
Merge
DAPI
Flag-B4GALT5
GP3-Myc
Merge
Flag-B4GALT5

## Slide 5
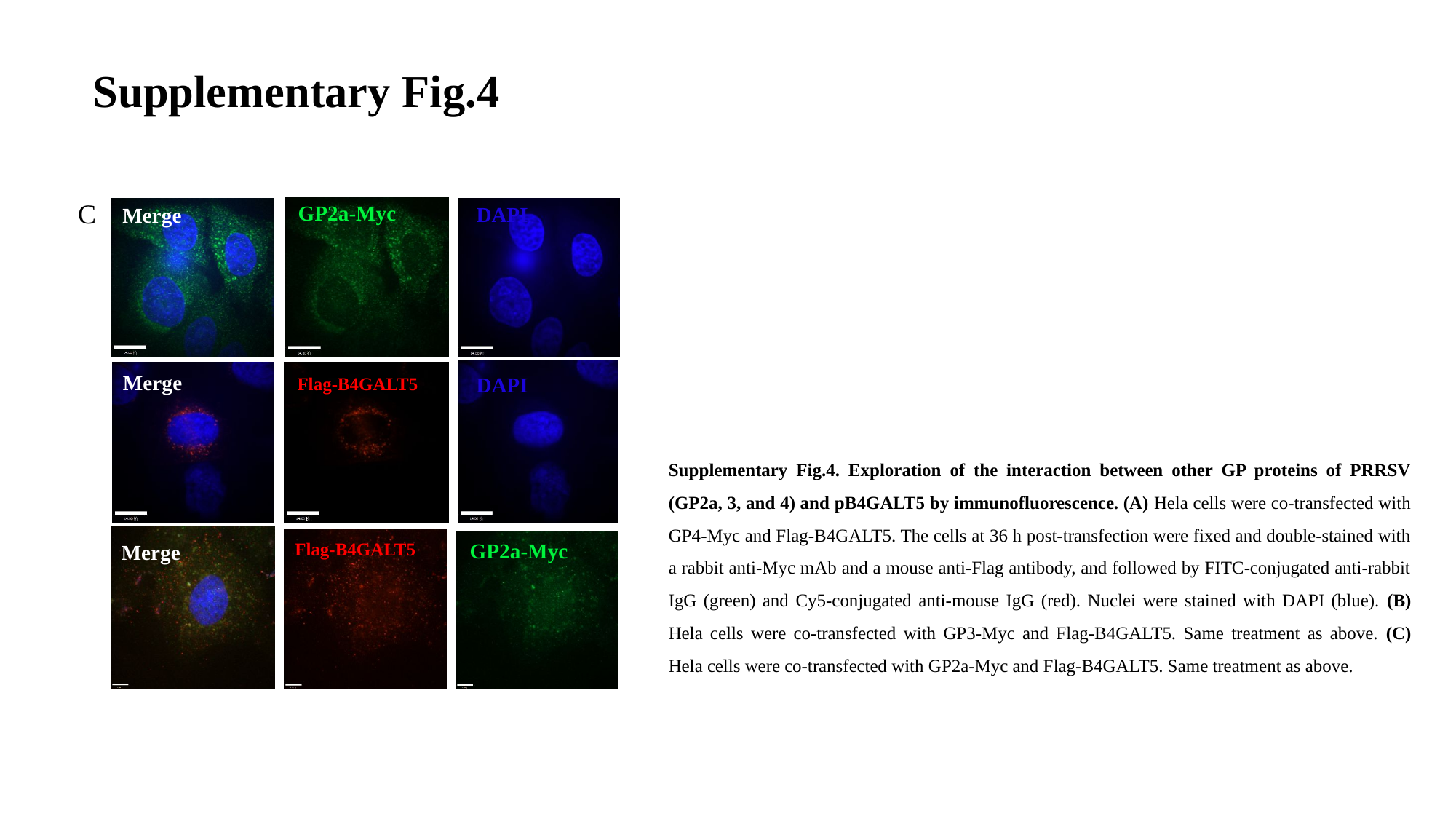

Supplementary Fig.4
C
GP2a-Myc
DAPI
Merge
Merge
DAPI
Flag-B4GALT5
Flag-B4GALT5
GP2a-Myc
Merge
Supplementary Fig.4. Exploration of the interaction between other GP proteins of PRRSV (GP2a, 3, and 4) and pB4GALT5 by immunofluorescence. (A) Hela cells were co-transfected with GP4-Myc and Flag-B4GALT5. The cells at 36 h post-transfection were fixed and double-stained with a rabbit anti-Myc mAb and a mouse anti-Flag antibody, and followed by FITC-conjugated anti-rabbit IgG (green) and Cy5-conjugated anti-mouse IgG (red). Nuclei were stained with DAPI (blue). (B) Hela cells were co-transfected with GP3-Myc and Flag-B4GALT5. Same treatment as above. (C) Hela cells were co-transfected with GP2a-Myc and Flag-B4GALT5. Same treatment as above.
